# Dual action effects of ethyl-p-methoxycinnamate against dengue virus infection and inflammation via NF-κB pathway suppression

**DOI:** 10.1038/s41598-024-60070-1

**Published:** 2024-04-23

**Authors:** Mayuri Tarasuk, Pucharee Songprakhon, Phunuch Muhamad, Aussara Panya, Pachara Sattayawat, Pa-thai Yenchitsomanus

**Affiliations:** 1 Graduate Program in Bioclinical Sciences, Chulabhorn International College of Medicine, Thammasat University, Pathum Thani, Thailand; 2 Division of Molecular Medicine, Research Department, and Siriraj Center of Research Excellence for Cancer Immunotherapy (SiCORE-CIT), Faculty of Medicine Siriraj Hospital, Mahidol University, Bangkok, Thailand; 3 Drug Discovery and Development Center, Office of Advanced Science and Technology, Thammasat University, Pathum Thani, Thailand; 4 Cell Engineering for Cancer Therapy Research Group, Faculty of Science, Chiang Mai University, Chiang Mai, Thailand

**Keywords:** Viral infection, Dengue virus

## Abstract

Dengue virus (DENV) infection can lead to severe outcomes through a virus-induced cytokine storm, resulting in vascular leakage and inflammation. An effective treatment strategy should target both virus replication and cytokine storm. This study identified *Kaempferia galanga* L. (KG) extract as exhibiting anti-DENV activity. The major bioactive compound, ethyl-p-methoxycinnamate (EPMC), significantly reduced DENV-2 infection, virion production, and viral protein synthesis in HepG2 and A549 cells, with half-maximal effective concentration (EC_50_) values of 22.58 µM and 6.17 µM, and impressive selectivity indexes (SIs) of 32.40 and 173.44, respectively. EPMC demonstrated efficacy against all four DENV serotypes, targeting the replication phase of the virus life cycle. Importantly, EPMC reduced DENV-2-induced cytokines (IL-6 and TNF-α) and chemokines (RANTES and IP-10), as confirmed by immunofluorescence and immunoblot analyses, indicating inhibition of NF-κB activation. EPMC's role in preventing excessive inflammatory responses suggests it as a potential candidate for dengue treatment. Absorption, distribution, metabolism, excretion, and toxicity (ADMET) and drug-likeness for EPMC were predicted using SwissADME and ProTox II servers, showing good drug-like properties without toxicity. These findings highlight KG extract and EPMC as promising candidates for future anti-dengue therapeutics, offering a dual-action approach by inhibiting virus replication and mitigating inflammatory reactions.

## Introduction

Dengue, caused by DENV, is a widespread mosquito-borne viral disease with four serotypes (DENV-1-4) that can lead to severe dengue, characterized by vascular leakage and shock, potentially fatal. Nearly half the world's population is at risk, with an estimated 390 million annual infections, 96 million symptomatic cases^1^. Currently, no effective vaccine or specific antiviral exists. Dengvaxia (CYD-TDV), the first licensed dengue vaccine, is not considered safe for dengue-naïve children^2^. Meanwhile, the promising new dengue vaccine, Qdenga (TAK-003), has received approval from the European Medicines Agency (EMA) but still requires additional safety and efficacy data for approval by the United States Food and Drug Administration (FDA)^3,4^. Especially, the efficacy-risk profile for DENV-3 and DENV-4 in seronegative individuals is currently under assessment. Thus, the urgent need persists for antiviral agents to hinder virus replication or mitigate DENV-induced immunopathology.

DENV's complex pathogenic mechanisms involve viral factors and host immune responses. DENV is a single-stranded positive-sense RNA virus with structural proteins (capsid [C], envelope [E], and membrane [M]) and nonstructural (NS) proteins (NS1, NS2A, NS2B, NS3, NS4A, NS4B, and NS5) involved in viral replication. Some DENV proteins induce inflammation by triggering the release of pro-inflammatory cytokines and chemokines, activating immune cells. The E protein domain III region triggers cytokine production via the NF-κB pathway^5^. NS1 disrupts endothelial cell integrity and induces cytokine production through macrophage and peripheral blood mononuclear cell activation^6^. The NS5 protein is crucial for DENV replication and modulating the host immune response. NS5 possesses enzymatic activities responsible for viral genome replication, including a methyltransferase (MTase) domain for capping and an RNA-dependent RNA polymerase (RdRp) domain for RNA synthesis. NS5 impairs IFN-α signaling, which is critical for innate antiviral responses during the early stages of infection^7^. On the other hand, NS5 induces the production of chemokines (IL-8, RANTES, MIP-1α, and MIP-1β), potentially promoting viral replication and inflammation in DENV infection^8^. A previous study demonstrated that NS5 triggers RANTES production through NF-κB signaling pathway^9^. NS5 translocates into the nucleus and interacts with the death-domain-associated protein (Daxx), resulting in the activation of RANTES expression.

Severe dengue is a life-threatening complication characterized by fluid leakage, which leads to pleural effusions, ascites, shock, and organ dysfunction. The most influential risk factor of severe dengue is a secondary infection by a different DENV serotype, which triggers a more severe immunological response in the patients^10^. Patients with severe dengue exhibit significantly elevated levels of various inflammatory cytokines and chemokines, including GM-CSF, IFN-γ, IL-10, IL-1β, IL-8, MCP-1, IL-6, MIP-1β, IP-10, TNF-α, and IL-18, compared to those with mild or moderate cases^11–16^. This uncontrolled immune response impairs virus clearance and contributes to vascular leakage and excessive inflammation. Based on these findings, we hypothesize that an effective anti-dengue drug should be capable of inhibiting both virus replication and the cytokine storm, which refers to the excessive release of immune signaling molecules.

Our hypothesis finds strong support in a prior study by Rattanaburee et al.^17^. They showed that the combination of the antiviral agent ribavirin (RV) with an anti-inflammatory compound (Compound A) significantly improved the effectiveness of reducing DENV replication and cytokine/chemokine production in DENV-infected cells. We have recently uncovered α-mangostin (α-MG), a compound that showcases dual capabilities against DENV infection, encompassing both antiviral and anti-inflammatory effects. It holds the potential to serve as a standalone agent, effectively curtailing DENV replication and mitigating the pathological immune response. This discovery offers a promising avenue for preventing severe dengue, as outlined in our study^18^. One limitation of α-MG is its low selectivity indexes (SIs), which stand at approximately 2.88 in HepG2 cells (unpublished data) and 1.28 in monocyte-derived dendritic cells (moDCs)^19^. The potential antiviral candidate compounds for further investigation typically necessitate an SI of at least 10 for consideration^20^. Hence, we are actively searching for new candidates that can achieve dual antiviral and anti-inflammatory effects while maintaining lower toxicity levels.

*Kaempferia galanga,* an aromatic ginger, thrives in India, China, Myanmar, Indonesia, Malaysia, and Thailand. The rhizome of *K. galanga* has a long history of use as a flavor spice and a traditional medicinal herb for treating various ailments, including colds, dry coughs, toothaches, digestive disorders, rheumatism, and hypertension^21^. Various compounds have been identified from the rhizome of *K. galanga*^21^. Gas chromatography–mass spectroscopy (GC–MS) analysis revealed that ethyl-p-methoxycinnamate (EPMC) emerges as the predominant bioactive constituent, constituting a substantial 94.87% of the total area composition in the ethanolic KG extract^22^. KG extracts and secondary metabolites of the herb exhibit numerous pharmacological properties, including anti-inflammatory, antioxidant, anticancer, antibacterial, and anti-angiogenic effects. KG extract significantly reduced inflammation in the carrageenan and cotton pellet granuloma models^23^. EPMC, identified by Umar et al.^24^, plays a pivotal role in anti-inflammatory effect of *K. galanga* by suppressing cyclooxygenase-1 (COX-1) and COX-2 enzymes. Additionally, Dwita et al. demonstrated that the EPMC isolate from *K. galanga* exhibits anti-inflammatory activity in two animal models—the carrageenan-induced granuloma air pouch model and the pleurisy model—by reducing both leukocyte numbers and leukotriene B4 (LTB4) concentrations^25^. Furthermore, EPMC exhibits significant anti-inflammatory potential by inhibiting pro-inflammatory cytokines, including IL-1 and TNF-α, as well as angiogenesis^26^. However, the existing studies provide limited information on the antiviral activity of either *K. galanga* or EPMC. A recent investigation discovered that KG extract exerts antiviral effects against pseudorabies virus (PrV), a herpesvirus causing severe diseases in mammals. Administering KG extract to PrV-infected mice improved their survival rates by reducing viral infection and severity. This treatment also regulated immune responses, impacting cytokine levels such as IFN-α, IFN-β, IL-4, IL-6, and TNF-α in the serum of PrV-infected mice^27^.

However, the therapeutic capabilities of KG extract and EPMC against DENV infection have not been fully investigated. This study aims to explore the anti-DENV potential of KG extract and examine the antiviral and anti-inflammatory effects of EPMC against DENV infection, elucidating the associated molecular mechanisms.

## Results

### *Kaempferia galanga *L. extract inhibited DENV-2 infection

**Figure 1:**
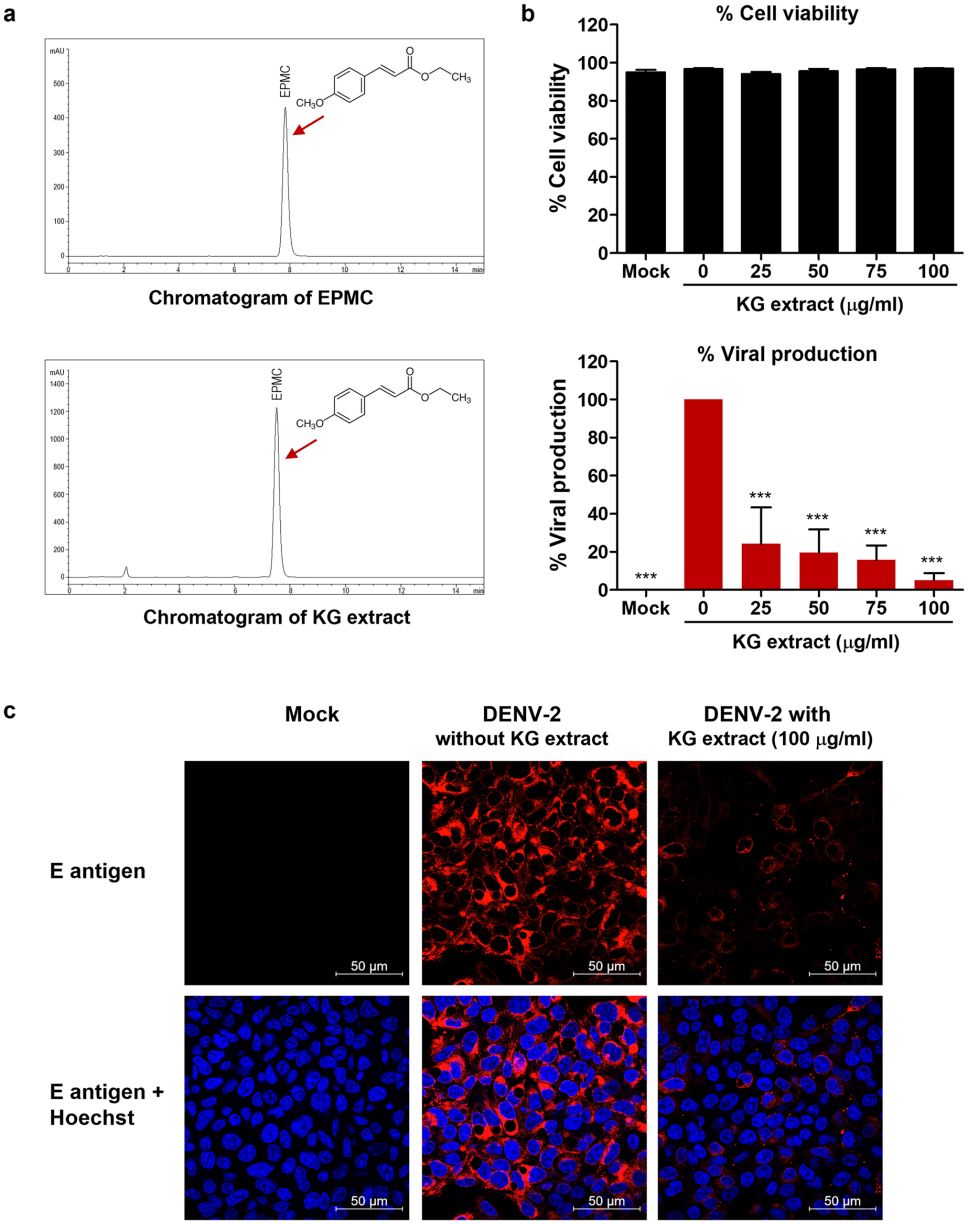
High-performance liquid chromatography (HPLC) profiles of KG extract and anti-DENV-2 effects in HepG2 cells. (**a**) HPLC chromatogram of EPMC standard compound (upper panel) and KG extract (lower panel). (**b**) Evaluation of the impact of KG extract on DENV-2 production, with cell viability percentages assessed through the trypan blue exclusion assay (upper panel) and DENV-2 production in culture supernatants determined via FFU assay (lower panel). The results are presented as mean ± SD from three independent experiments. Statistical differences, compared to the control group, were calculated using one-way ANOVA and Tukey’s HSD test (*p < 0.05, **p < 0.01, ***p < 0.001). (**c**) Immunofluorescence assay detecting DENV-2 E protein (in red), with nuclear staining using Hoechst (in blue). Representative images from three independent experiments are shown.

To evaluate the effectiveness of *K. galanga* L. against DENV-2, we prepared an ethanolic extract from the rhizomes of *K. galanga* L. This extract had a semi-solid, oily consistency, exuding a pleasant fruity aroma and displaying an amber hue. High-performance liquid chromatography (HPLC) analysis of the KG extract unveiled a distinct peak with the same retention time as EPMC (7.83 min), a known bioactive marker compound, accounting for 95.38% of the total peak area (Fig. 1a). In order to gauge the impact of KG extract on HepG2 cells, we conducted cytotoxicity assessments, yielding a CC_50_ value of 141.46 ± 4.45 μM. Subsequently, we exposed HepG2 cells to KG extract concentrations ranging from 25 to 100 μg/μl following a 2-h DENV-2 infection. After 24 h post-infection (hpi), we observed a consistent cell viability of at least 94% across all tested conditions, signifying the non-toxic nature of these conditions for HepG2 cells. Notably, treatment with KG extract at concentrations of 25, 50, 75, and 100 μg/μl significantly reduced virus production to 24.16%, 19.48%, 15.69%, and 5.08%, respectively (Fig. 1b). Furthermore, our immunofluorescence results underscored the anti-DENV-2 activity of KG extract, with treatment at 100 μg/μl notably reducing the number of infected cells (Fig. 1c).

### EPMC reduced DENV infection, virus production, and viral protein synthesis

**Figure 2:**
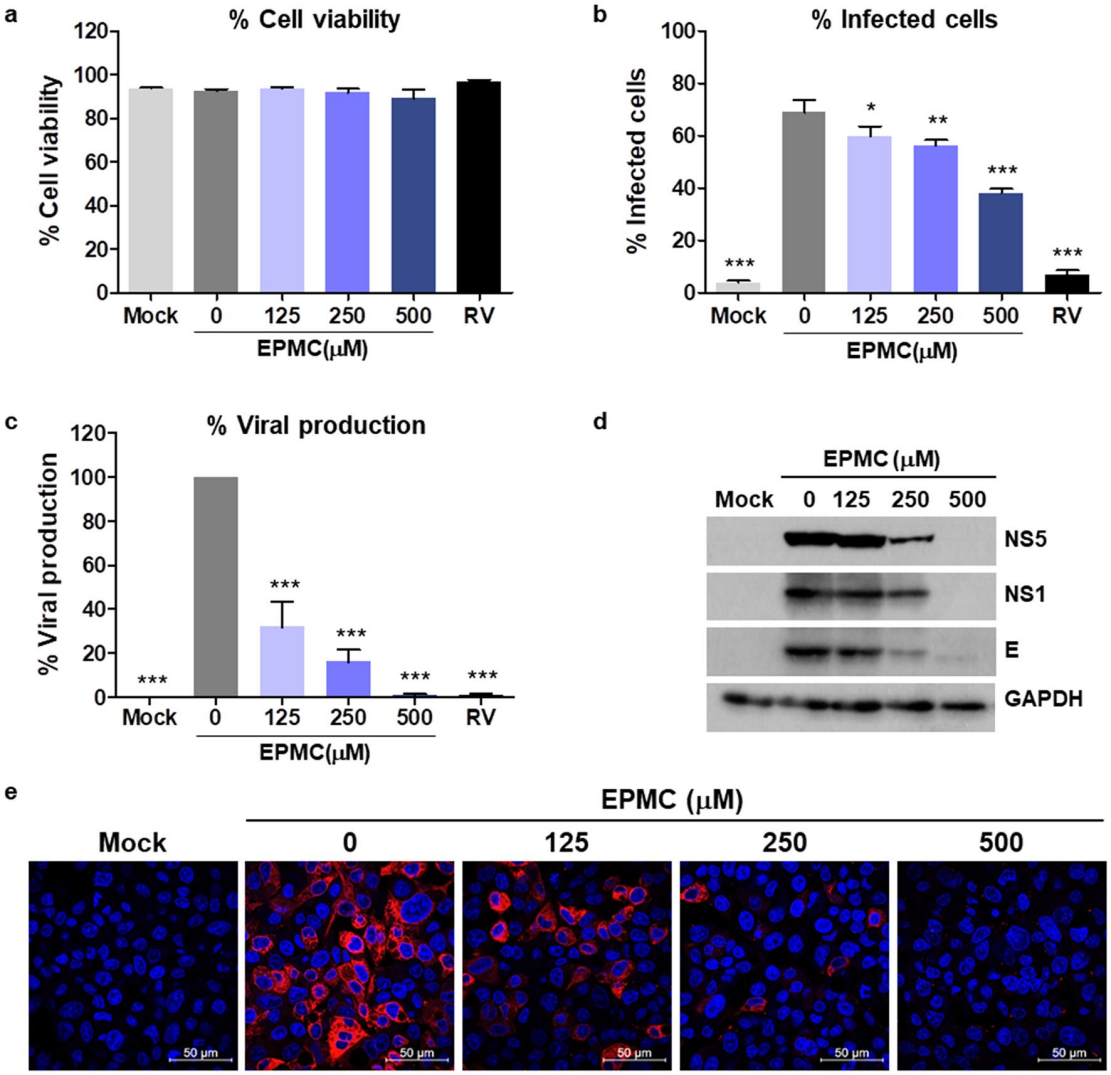
EPMC attenuates DENV-2 infection, progeny virus production, and protein expression in HepG2 cells. HepG2 cells were infected with DENV-2 (MOI of 5) and treated with either EPMC (0–500 μM) or RV (100 μM) for 24 h. (**a**) Assessment of cell viability, expressed as percentages, through the trypan blue exclusion assay. (**b**) Determination of the proportion of infected cells via flow cytometry. (**c**) Quantification of progeny virus production in culture supernatants using the FFU assay. The results are presented as mean ± SD from three independent experiments. Statistical differences, compared to the control group, were calculated using one-way ANOVA and Tukey’s HSD test (*p < 0.05, **p < 0.01, ***p < 0.001). (**d**) Immunoblot analysis revealing DENV-2 NS5, NS1, and E protein levels in lysates of infected cells. The original blots are presented in Supplementary Figure S1. (**e**) Visualization of the DENV-2 E protein (in red) through immunofluorescence assay, accompanied by nuclear staining using Hoechst (in blue).

We proceeded to assess both the cytotoxicity and antiviral potential of EPMC, given its status as the primary component of the KG extract. We evaluated the impact of EPMC on the cytotoxicity HepG2 cells using the PrestoBlue assay, yielding the maximum-non-toxic dose (MNTD_80_) and the half-maximal cytotoxicity concentration (CC_50_) values of 602.68 ± 56.54 μM and 731.51 ± 38.85 μM, respectively (Table 1). To gauge the antiviral efficacy of EPMC, we quantified the number of DENV-2-infected cells via flow cytometry. After 24 hpi, we observed over 89% cell viability across all conditions, indicating no cytotoxicity of EPMC and RV to HepG2 cells (Fig. 2a). EPMC demonstrated a dose-dependent reduction in infected cells. Specifically, at concentrations of 125, 250, and 500 µM, EPMC decreased the percentage of DENV-2-infected HepG2 cells to 60%, 56%, and 38%, respectively, compared to the untreated control, which exhibited 69% infected cells (Fig. 2b). We also assessed the effect of EPMC on virus production using the FFU assay. In comparison to untreated DENV-2-infected cells, defined as having 100% virus production, treatment with EPMC at 125, 250, and 500 µM significantly reduced virus production to 32%, 16%, and 1%, respectively (Fig. 2c). Furthermore, RV treatment substantially decreased infected cells to 7% and virus production to 1%. Based on the reduction in virus foci, the half-maximal effective concentration (EC_50_) value of EPMC in DENV-2-infected HepG2 cells was determined to be 22.58 ± 3.67 μM, with an SI value of 32.40 (Table 1 and Fig. S4). We also assessed the impact of EPMC exposure on DENV-2 protein production (NS5, NS1, and E proteins) through immunoblot analysis, revealing a clear, dose-dependent reduction compared to untreated infected cells (Fig. 2d). Additionally, immunofluorescence results confirmed the anti-DENV-2 activity of EPMC in HepG2 cells, showing a significant decrease in DENV E-protein within infected cells (Fig. 2e).

**Table 1:** Cytotoxicity assessment of EPMC in HepG2 and A549 cells, and evaluation of EPMC's anti-DENV-2 activities.

Cell	MNTD_80_ (μM) ± SD	CC_50_ (μM) ± SD	EC_50_ (μM) ± SD	SI
HepG2	602.68 ± 56.54	731.51 ± 38.85	22.58 ± 3.67	32.40
A549	851.61 ± 99.00	1070.13 ± 51.86	6.17 ± 0.28	173.44

The maximum-non-toxic dose (MNTD_80_) is the dose at which 80% of the cells remain viable, while the half-maximal cytotoxicity concentration (CC_50_) is the concentration at which 50% of the cell viability is reduced. The half-maximal effective concentration (EC_50_) refers to the concentration of EPMC required to inhibit viral progeny by 50%. The selectivity index (SI) was calculated as the ratio of EPMC's cytotoxicity to its antiviral activity (CC_50_/EC_50_). Data were acquired from three independent experiments and presented as mean ± SD.

Furthermore, to validate the anti-DENV activity of EPMC, we utilized A549 cells, known for their high susceptibility to DENV infection. Cytotoxicity assessments in A549 cells yielded MNTD_80_ and CC_50_ values of 851.61 ± 99.00 μM and 1070.13 ± 51.86 μM, respectively (Table 1). Treatment of DENV-2-infected A549 cells with EPMC led to a substantial reduction in DENV-2 infection, virus production, and viral protein synthesis (Fig. S2). The EC_50_ value of EPMC in DENV-2-infected A549 cells was determined to be 6.17 ± 0.28 μM, with an SI value of 173.44 (Table 1 and Fig. S4).

**Figure 3:**
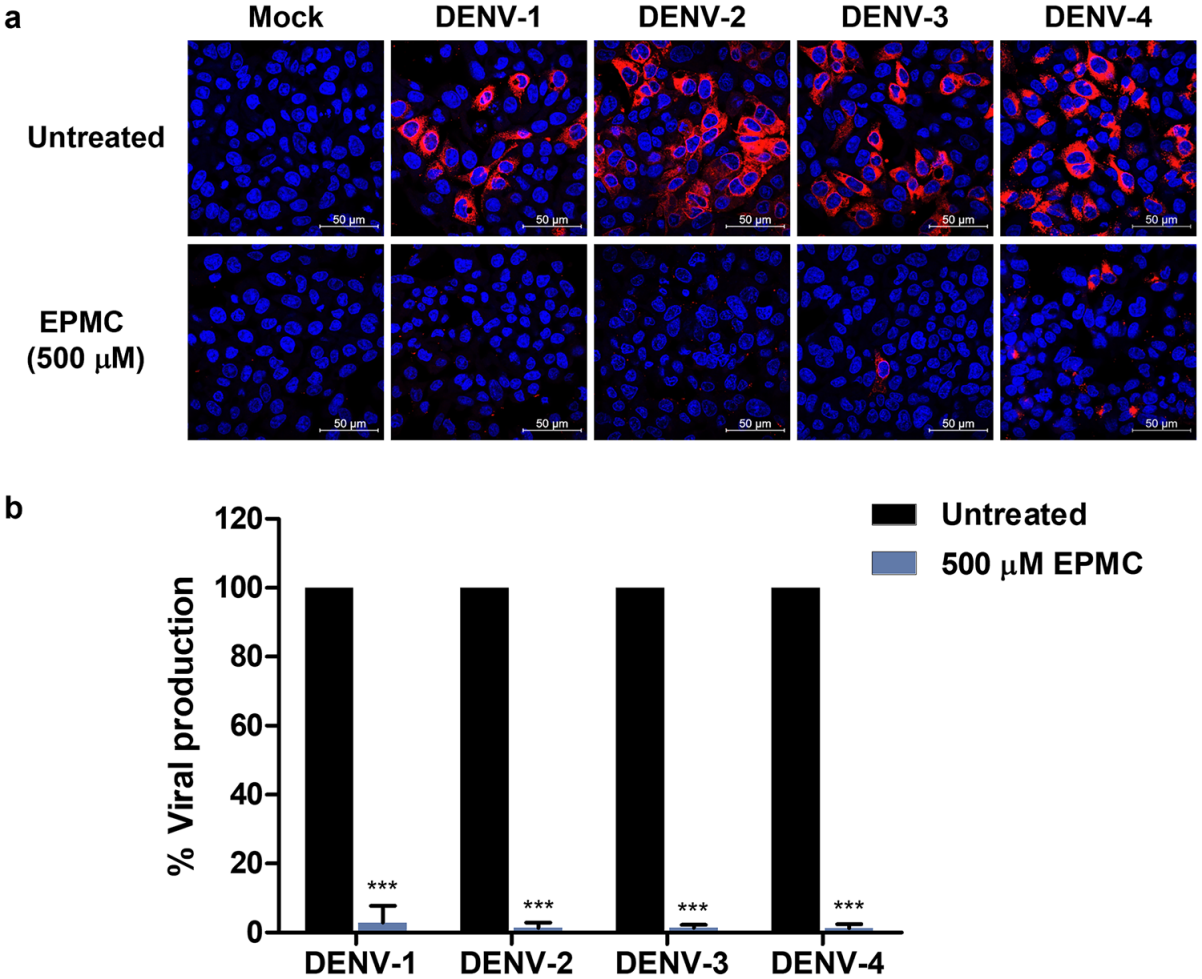
Antiviral activity of EPMC against DENV serotypes 1, 2, 3, and 4. HepG2 cells were infected with either DENV-1, DENV-2, DENV-3, or DENV-4 at MOI 5, 5, 10, and 5, respectively, and treated with EPMC (500 μM) for 24 h. (**a**) Immunofluorescence assay demonstrating the detection of DENV-2 E protein (in red), with nuclear staining performed using Hoechst (in blue). (**b**) Quantification of DENV production in culture supernatants via FFU assay. An unpaired Student's t-test was employed to compare virus production levels between EPMC-treated and untreated DENV-infected HepG2 cells (***p < 0.001).

To further ascertain whether EPMC could inhibit DENV infection across other serotypes, we infected HepG2 cells with DENV-1, DENV-2, DENV-3, or DENV-4 and subsequently treated them with 500 µM of EPMC. We detected the E protein in the infected cells using an immunofluorescence assay and quantified infectious virions in the culture supernatant using the FFU assay. EPMC treatment distinctively reduced the number of DENV-infected cells across all four serotypes and significantly decreased virus production in culture supernatants (Fig. 3).

### EPMC inhibited both the early and late stages of the DENV life cycle

**Figure 4:**
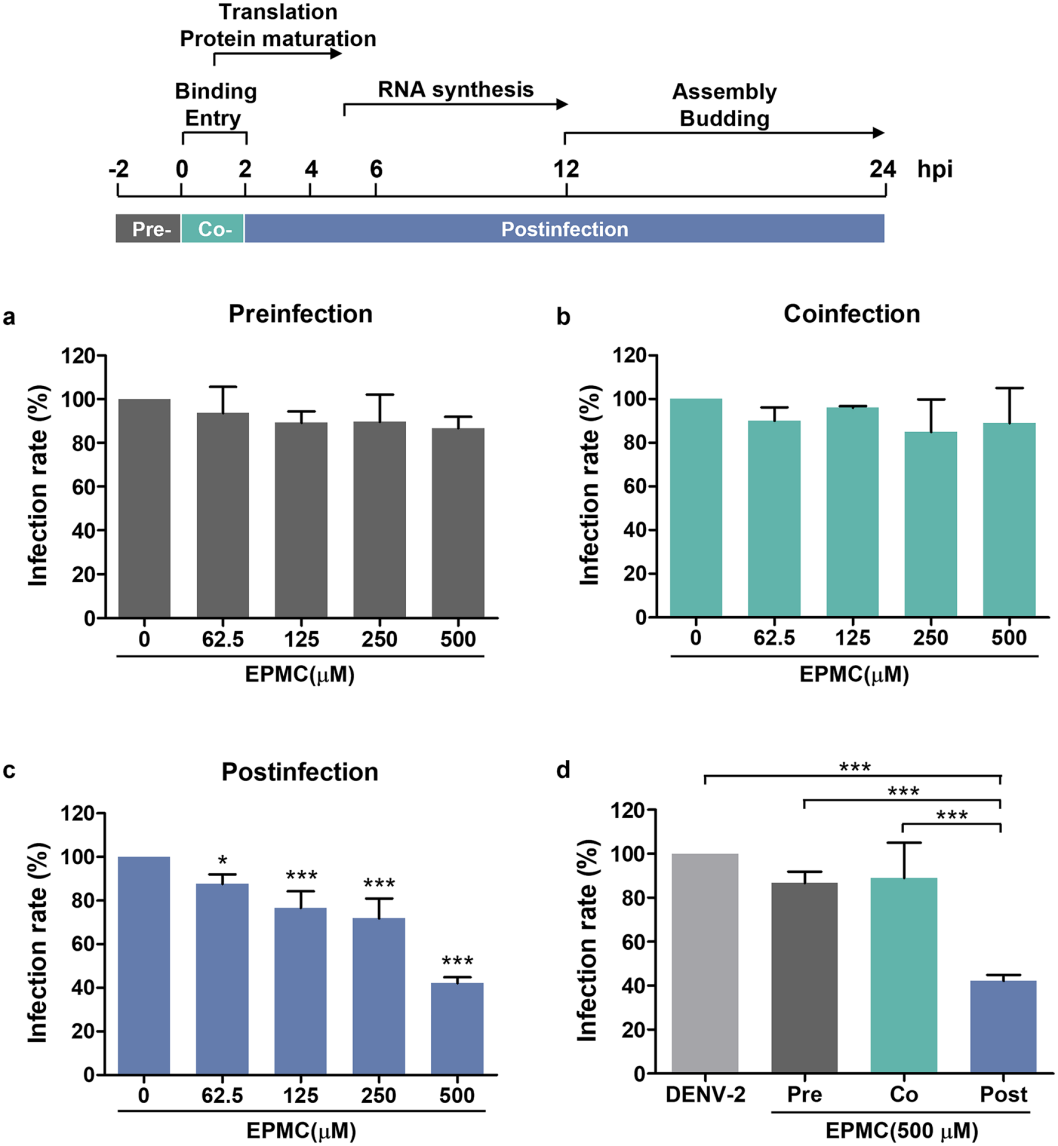
EPMC inhibits DENV-2 infection at the postinfection stage. HepG2 cells were treated with different concentrations of EPMC (0–500 μM) at preinfection, coinfection, or postinfection of DENV-2 (MOI of 5). At 24 hpi, the percentages of infected cells were evaluated by flow cytometry. (**a**–**c**) Influence of EPMC on the proportions of infected cells in preinfection, coinfection, or postinfection scenarios. The percentage of infected cells in the untreated control group was set as 100%. (**d**) Comparative assessment of the inhibitory effects of EPMC at 500 µM across various additional steps. The results are presented as mean ± SD from three independent experiments. Statistical differences, compared to the untreated control group, were calculated using one-way ANOVA and Tukey’s HSD test (*p < 0.05, **p < 0.01, ***p < 0.001).

A time-of-drug-addition assay was conducted to elucidate the specific stage of the DENV life cycle targeted by EPMC in exerting its antiviral activity. HepG2 cells were infected with DENV-2 and subsequently subjected to treatment with EPMC at concentrations ranging from 62.5 to 500 μM at distinct time points, encompassing preinfection, coinfection, and postinfection phases. The findings unequivocally revealed that the administration of EPMC led to a significant reduction in the proportion of infected cells exclusively under the postinfection condition. Conversely, treatment with EPMC during the preinfection and coinfection phases exhibited no discernible impact (Fig. 4a–c). A comparative assessment of the effects of EPMC at the concentration of 500 μM across different conditions demonstrated that postinfection treatment with EPMC culminated in a reduction of infected cells to 42%, in stark contrast to the preinfection and coinfection conditions, where 87% and 89% of cells remained infected, respectively (Fig. 4d).

**Figure 5:**
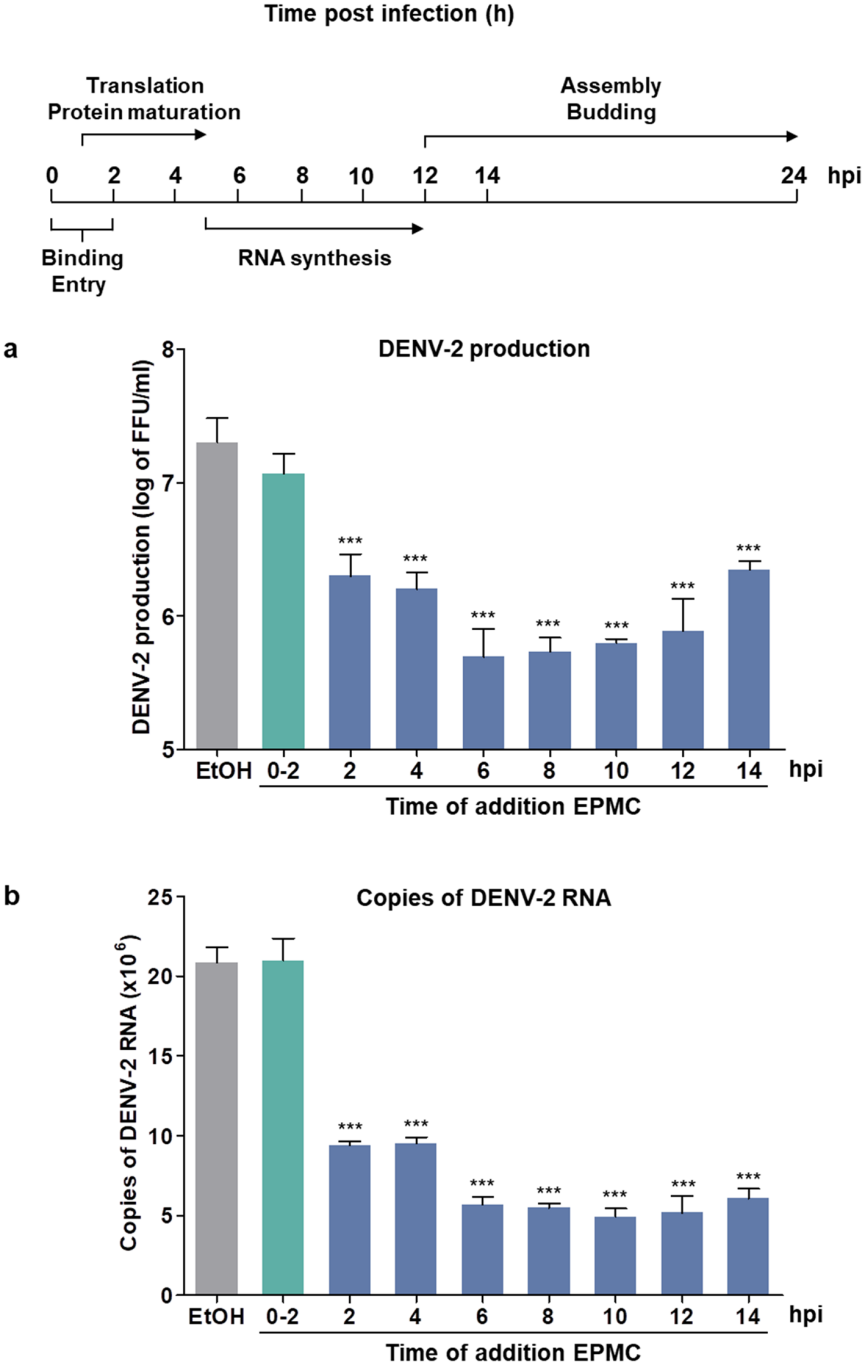
EPMC affects both the early and late stages of DENV replication. HepG2 cells were infected with DENV-2 (MOI of 5) and EPMC (500 μM) were added at 0–2, 2, 4, 6, 8, 10, 12, and 14 hpi. At 24 hpi, (**a**) the virus titers in the culture supernatants were quantified by FFU assay and (**b**) the intracellular viral RNA levels were quantified by qRT-PCR. The results are presented as the mean ± SD from three independent experiments. Statistical differences, compared to the untreated control group, were calculated using one-way ANOVA and Tukey’s HSD test (***p < 0.001).

In an endeavor to pinpoint the precise stage during the postinfection phase at which EPMC exerts its antiviral influence, HepG2 cells were infected with DENV-2 and subsequently treated with 500 μM EPMC commencing at 2, 4, 6, 8, 10, 12, and 14 hpi until the supernatants and cell lysates were harvested at 24 hpi. Analysis of infectious progeny released in the supernatant revealed that the progeny titer remained unchanged when EPMC was added simultaneously with DENV-2 infection for 2 h, suggesting that EPMC does not influence DENV entry. At post viral entry, EPMC not only affected an early stage of the viral life cycle but also diminished progeny titers when added at later stages. Specifically, the infectious virus production was significantly reduced when EPMC was added at 2, 4, 6, 8, 10, 12, and 14 hpi (Fig. 5a). The maximum reduction in virus production was observed when EPMC was added at 6–10 hpi, whereas its efficacy notably diminished at 12 and 14 hpi, suggesting that EPMC is highly effective at the RNA synthesis step of DENV life cycle (Fig. 5a). Similarly, EPMC treatment was maximally effective in reducing intracellular viral RNA when added at 6–10 hpi, while the inhibitory effect slightly decreased when EPMC was added at later time points (12 and 14 hpi), suggesting that EPMC interferes with RNA replication and virion biogenesis (Fig. 5b). Thus, the data together indicate that EPMC possibly inhibits viral RNA replication at an early step of the DENV life cycle, but it also affects a late stage of virion biogenesis, such as assembly and budding. This is supported by a strong antiviral effect consistently observed in infected cells with EPMC treatment starting as late as 12 and 14 hpi.

### EPMC reduced cytokine and chemokine transcription and secretion induced by DENV-2 infection

**Figure 6:**
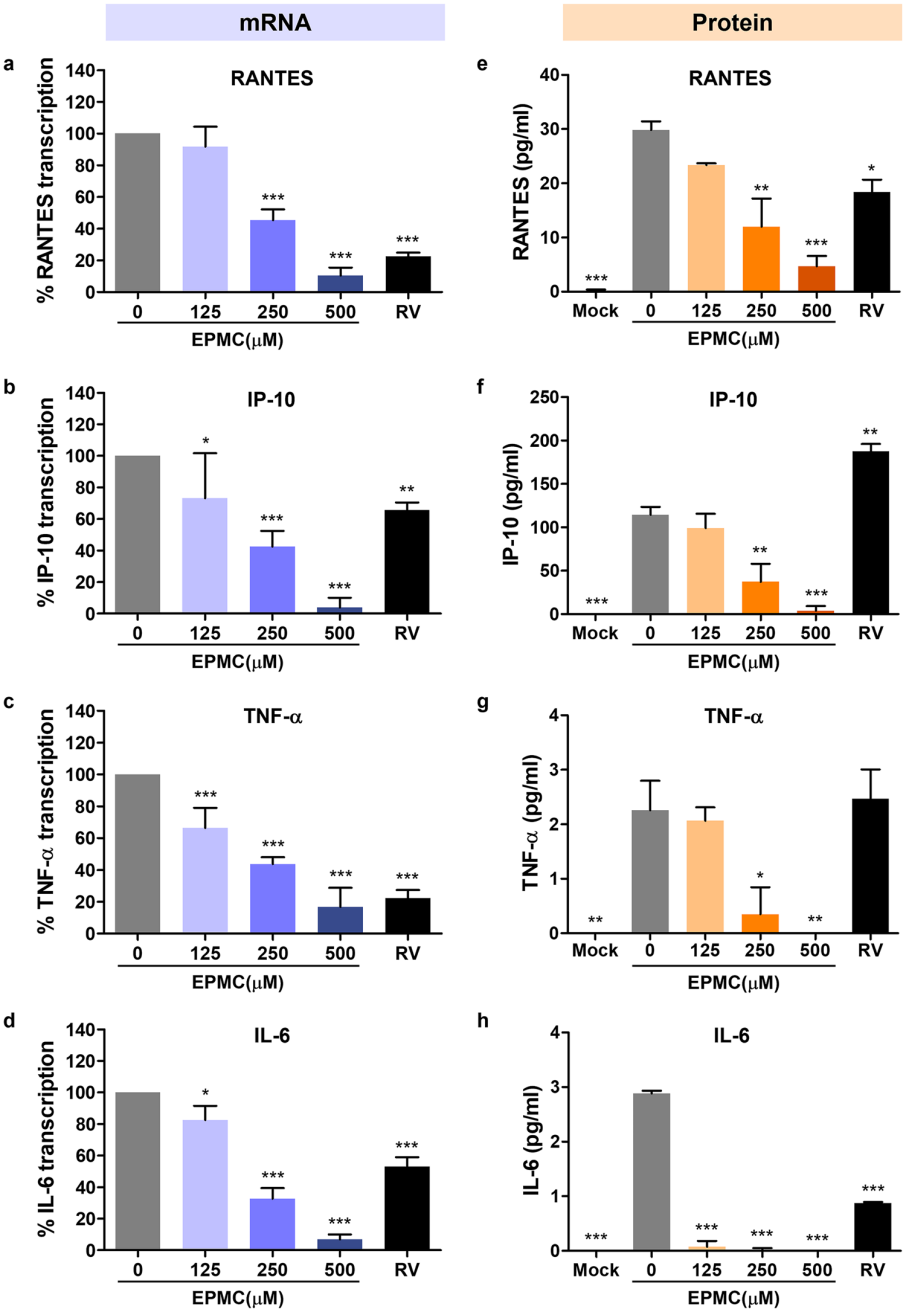
EPMC reduces cytokine and chemokine production in DENV-infected HepG2 cells. HepG2 cells were infected with DENV-2 (MOI of 5) and treated with either EPMC (0–500 μM) or RV (100 μM) for 24 h. (**a**–**d**) The mRNA levels of RANTES, IP-10, TNF-α, and IL-6 in DENV-2-infected HepG2 cells were determined by qRT-PCR. The transcription level of each cytokine/chemokine in untreated DENV-infected cells was defined as 100%. (**e**–**h**) The cytokine/chemokine levels of RANTES, IP-10, TNF-α, and IL-6 in the culture supernatants were determined by flow cytometry using multiplexed bead-based immunoassays. The results are presented as the mean ± SD from three independent experiments. Statistical differences, compared to the untreated control group, were calculated using one-way ANOVA and Tukey’s HSD test (*p < 0.05, **p < 0.01, ***p < 0.001).

The impact of EPMC on the production of cytokines and chemokines induced by DENV was assessed at both mRNA expression and secreted protein levels. Specifically, the cytokines and chemokines RANTES, IP-10, TNF-α, and IL-6 were chosen as representative markers based on prior investigations^18^. HepG2 cells were subjected to DENV-2 infection and subsequently treated with either EPMC or ribavirin (RV). Following a 24-h post-infection period, cellular harvesting was performed to quantify the mRNA levels of cytokine and chemokine genes utilizing qRT-PCR, while the culture supernatants were collected for the quantification of secreted cytokine and chemokine levels via multiplexed bead-based immunoassays. Comparatively, the expression levels of RANTES, IP-10, TNF-α, and IL-6 were observed to be elevated in DENV-2-infected cells when compared to mock-infected cells. The presented data are depicted as a percentage relative to the transcription levels in untreated DENV-2-infected cells, which were set as 100%. Intriguingly, treatment with EPMC resulted in a significant and dose-dependent reduction in the transcription of all examined cytokine and chemokine genes. Conversely, treatment with RV also led to a decrease in the transcription of these genes, albeit to a lesser extent than observed with a concentration of 500 μM of EPMC (Fig. 6a–d). Moreover, we tested the effect of EPMC on cytokine/chemokine expression in mock-infected HepG2 cells, and the results confirmed that it had no effect on the cytokine/chemokine profile in the uninfected cells (Fig. S5). In alignment with its impact on cytokine and chemokine transcription, EPMC exhibited a significant and dose-dependent reduction in the secretion of RANTES, IP-10, TNF-α, and IL-6 proteins compared to untreated DENV-2-infected cells (Fig. 6e–h). Conversely, treatment with RV resulted in significant reductions in the levels of RANTES and IL-6 (Fig. 6e, h), while the levels of IP-10 and TNF-α remained unaltered (Fig. 6f, g).

### EPMC suppressed DENV-2-induced NF-κB activation

**Figure 7:**
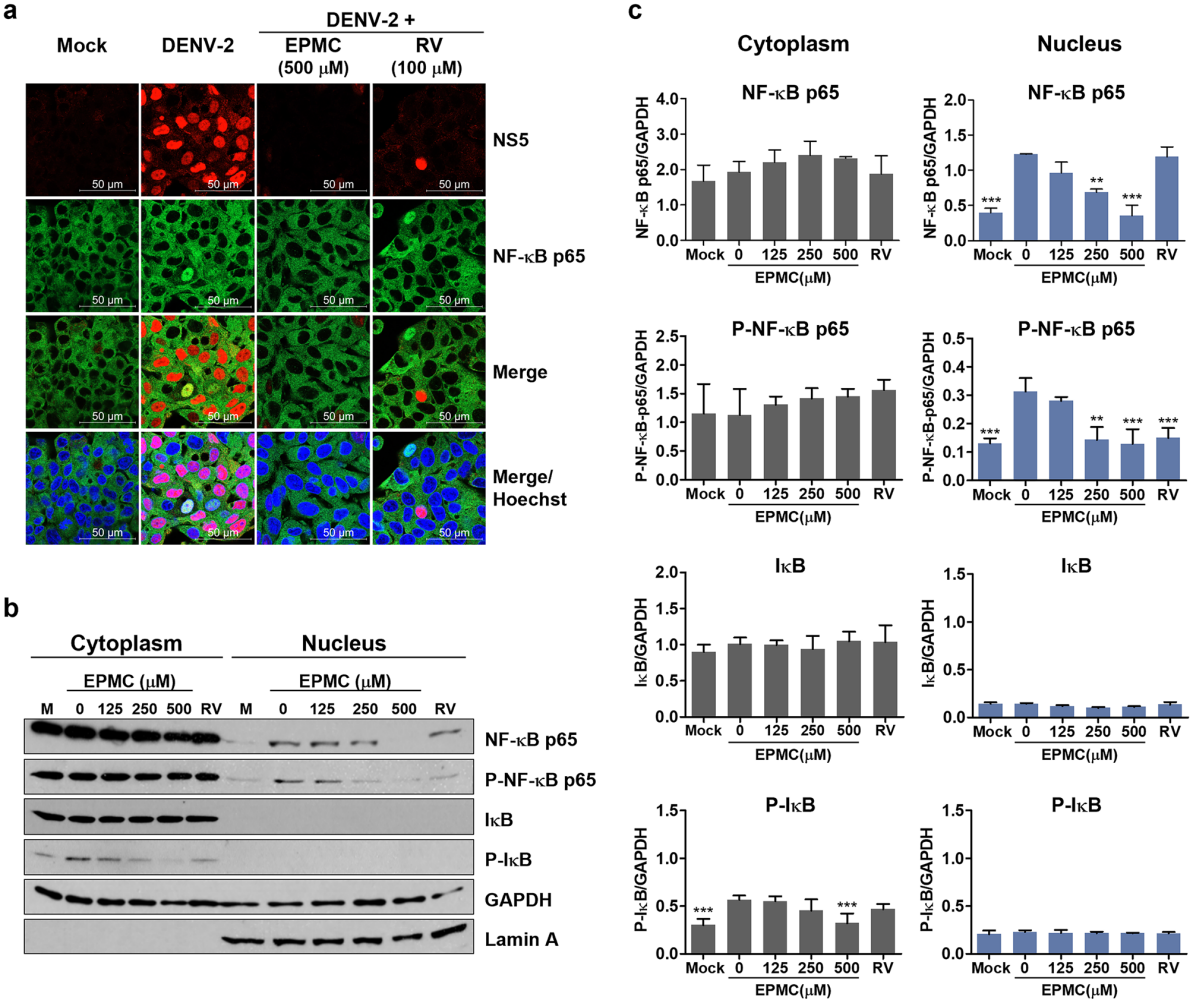
EPMC inhibits NF-κB nuclear translocation and suppresses DENV-induced NF-κB activation. HepG2 cells were infected with DENV-2 (MOI of 5) and treated with either EPMC (0–500 μM) or RV (100 μM) for 24 h. (**a**) The immunofluorescence assay illustrates the expression and intracellular localization of DENV NS5 (in red) and NF-κB p65 (in green). Nuclear staining using Hoechst dye is depicted in blue. (**b**) Cytoplasmic and nuclear proteins were fractionated. Immunoblot analysis reveals the expression of NF-κB p65, P-NF-κB p65, IκBα, and P-IκBα proteins. GAPDH was used as an internal control, and lamin A was used as a nuclear protein marker. (**c**) Protein levels of NF-κB p65, P-NF-κB p65, IκBα, and P-IκBα proteins were quantified using ImageJ software and normalized to the level of the GAPDH protein. The results are presented as the mean ± SD from three independent experiments. Statistical differences, compared to the untreated control group, were calculated using one-way ANOVA and Tukey’s HSD test (**p < 0.01, ***p < 0.001). The original blots are presented in Supplementary Figures S7, S8.

To gain deeper insights into the underlying mechanisms governing the antiviral and anti-inflammatory properties of EPMC, an investigation was carried out to assess the presence of NS5 and NF-κB p65 within DENV-2-infected cells employing an immunofluorescence assay. HepG2 cells were subjected to DENV-2 infection, followed by treatment with either EPMC or RV. In untreated DENV-2-infected cells, NS5 was predominantly localized within the cell nucleus, where it is hypothesized to play a role in suppressing host antiviral responses. Nuclear translocation of NF-κB p65 was observed in specific DENV-2-infected cells, a phenomenon absent in the mock control. Notably, EPMC treatment resulted in a noticeable reduction in the nuclear accumulation of both NS5 and NF-κB p65 in DENV-2-infected cells (Fig. 7a). Furthermore, the discernible impact of EPMC in diminishing nuclear NS5 presence and inhibiting the translocation of NF-κB p65 into the nucleus was also evident in DENV-2-infected A549 cells (Fig. S6).

To further delve into the influence of EPMC on DENV-2-induced NF-κB activation, an assessment of proteins associated with the NF-κB signaling pathway was conducted through immunoblot analysis in DENV-2-infected HepG2 cells. In the context of DENV-2 infection, both nuclear NF-κB p65 and phosphorylated NF-κB p65 (P-NF-κB p65) expression levels were significantly heightened compared to mock-infected cells. Concomitantly, a notable increase in cytoplasmic phosphorylated IκB (P-IκB) expression was observed. In stark contrast, treatment with EPMC at concentrations of 250 and 500 μM resulted in a significant reduction in nuclear levels of NF-κB p65 and P-NF-κB p65. Additionally, EPMC treatment at 500 μM elicited a significant decrease in cytoplasmic P-IκB expression. Conversely, treatment with RV only led to a significant reduction in nuclear P-NF-κB p65 expression (Fig. 7b,c). The nuclear marker, lamin A, which was exclusively localized in the nuclear fraction, confirmed the effective separation of the nucleus and cytoplasm during subcellular fractionation.

### Assessment of drug-likeness and ADMET of EPMC

The ADME analysis by the Swiss ADME webserver^28^ revealed physicochemical properties and drug likeness of EPMC. The laws of Lipinski’s^29^, Egan’s^30^, and Veber’s^31^ determine the properties of a drug as follows: molecular weight (MW) < 500, topological surface area (TPSA) < 140, number of H-bond acceptors (nOHNH) ≤ 5, number of H-bond donors (nON) ≤ 5, water partition coefficient (WLOGP) ≤ 5.88, and number of rotatable bonds (nrotb) ≤ 10^32^. EPMC passed the Lipinski’s, Egan’s, as well as Veber’s rules, indicating that it possesses good drug-like properties (Table S1) and exhibits good human gastrointestinal absorption (HIA), blood–brain barrier (BBB) permeation, and bioavailability. Moreover, the webserver ProTox-II^33^ was used to predict the toxicity properties of EPMC. Prediction of Toxicity Class of chemical divided into: Class 1: Fatal (LD_50_ ≤ 5 mg/kg), Class 2: Fatal (5 < LD_50_ ≤ 50 mg/kg), Class 3: Toxic (50 < LD_50_ ≤ 300 mg/kg), Class 4: Harmful (300 < LD_50_ ≤ 2000 mg/kg), Class 5: Maybe harmful (2000 < LD_50_ ≤ 5000 mg/kg), Class 6: Non-toxic (LD_50_ > 5000 mg/kg)^34^. EPMC was classified as a non-toxic compound with LD_50_ values of 7900 mg/kg. Furthermore, EPMC is anticipated to demonstrate no hepatotoxicity, cytotoxicity, carcinogenicity, mutagenicity, or immunotoxicity, as illustrated in Table S2. This suggests that EPMC is safe for biological administration and holds promise as a potential anti-DENV drug.

### Molecular docking of EPMC with DENV protein targets

The interaction between EPMC and DENV proteins can disrupt their function, thereby contributing to its antiviral activity. To test this hypothesis, we performed the molecular docking to investigate the ability of EPMC to interact with key DENV proteins involved in viral replication, including NS1, NS3, and NS5 proteins. We investigated the binding interaction of EPMC and DENV NS1 protein (PDB entry 4O6B). The result shows a weak interaction with the ChemPLP score of 39.23, whereas deoxycalyxin A reference ligand achieved 76.64. The interaction of EPMC contributed via hydrogen bond to VAL84 and LYS85, in addition to the pi-alkyl hydrophobic interaction to HIS129 (Fig. S9a). NS5 is a bi-functional enzyme with a methyltransferase (MTase) and RNA-dependent RNA polymerase (RdRp) activities. The crystal structures of these two functional domains were used as templates for docking. The docking pose of EPMC and MTase (PDB entry 1R6A) achieved the score of 43.00 (reference ligand achieved 76.98). The interaction formed via hydrophobic interactions of LYS105, VAL132, PHE133, and ILE147 with the ligand (Fig. S9b). The poses of EPMC and RdRp (PDB entry 5JJR) achieved the score of 46.05 (reference ligand achieved 99.33) through the hydrophobic interactions of LEU511, ARG737, MET761, MET765, ALA799, and TRP803 (Fig. S9c). Moreover, we investigated the interaction between EPMC and NS2B-NS3 protease. The NS2B-NS3 protease (PDB entry 4M9K) was found to interact with a score of 45.40 (reference ligand achieved 69.22) via the interactions of ILE1036, VAL1052, ARG1054, and ALA1056 with the ligand (Fig. S9d). Additionally, the NS3 helicase domain (PDB entry 2BHR) was found to interact with EPMC via hydrophobic interactions of ILE365 and PRO543, with the fitness score of 40.98 (reference ligand achieved 71.16) (Fig. S9e).

## Discussion

The escalating incidence and geographical spread of dengue cases highlights the urgent need for effective vaccines and therapeutics to combat this disease. Natural products have emerged as a vital reservoir for drug development due to their substantial diversity and low incidence of adverse effects. In this study, we present, for the first time, evidence of the anti-DENV activity of the *K. galanga* L. (KG) extract, as well as its principal bioactive compound, ethyl-p-methoxycinnamate (EPMC). Our investigation revealed that the KG extract exhibited significant anti-DENV-2 activity. EPMC was identified as the predominant component in our KG extract, constituting 95.38% of the total area composition, in line with findings from prior research^35^. Consequently, EPMC was selected for subsequent experiments to identify the active compound responsible for the anti-DENV activity in *K. galanga* L. Our results unequivocally demonstrate the potent antiviral effects of EPMC against DENV-2 in both HepG2 and A549 cells. HepG2 cells represent hepatic cells, whereas A549 cells represent lung cells—both being sites of DENV replication in infected humans^36^. Additionally, these two cell types exhibit elevated expression of cytokines and chemokines such as IL-6 and RANTES, which align with the heightened levels of these molecules observed in severe dengue patients^37,38^. EPMC treatment significantly reduces DENV-infected cells, progeny virus production, and viral protein synthesis in both cell types, underscoring the persistent nature of this anti-DENV effect across diverse cell types (Figs. 2 and S2). Remarkably, EPMC displayed no discernible toxicity towards either HepG2 or A549 cells, manifesting impressive selectivity indexes (SIs) of 32.40 and 173.44, respectively. In comparison to our previous study, the anti-DENV activity of EPMC in HepG2 cells exhibited a higher SI than α-mangostin (α-MG), suggesting the promising potential of EPMC as a candidate for combating DENV infections. Crucially, EPMC demonstrated broad-spectrum antiviral activity against all four DENV serotypes (Fig. 3), rendering it particularly valuable in addressing DENV infections in regions where all four serotypes are in circulation. Furthermore, other phytoconstituents of *K. galanga*, such as kaempferide and kaempferol, were examined to assess their anti-DENV-2 activity. Kaempferide exhibited a less pronounced reduction in virus production compared to EPMC, while kaempferol demonstrated no discernible effect (unpublished data).

As the precise mechanism underlying EPMC's action against DENV-2 remained enigmatic, we conducted time-of-drug-addition assays to dissect its impact on the DENV life cycle. Our findings revealed that EPMC effectively impeded DENV-2 infection at the post-entry stage without discernibly affecting DENV-2 binding and/or entry processes (Fig. 4). Further insights from time-of-drug-addition experiments pinpointed that EPMC notably curtailed virus production and intracellular viral RNA when introduced at 6–10 hpi, while the inhibitory effect of EPMC exhibited a diminishment when introduced at 12 and 14 hpi (Fig. 5). Noteworthy, significant reductions in virus production and intracellular viral RNA were observed at both the early and late stages of the DENV life cycle. These findings collectively suggest that EPMC exerts its anti-DENV-2 effects at multiple steps of viral intracellular replication. However, this experiment was conducted without synchronizing the cells before DENV infection. Thus, synchronizing them before conducting the time-of-drug-addition assay would better pinpoint the exact stage of the virus life cycle. In light of these observations, it is conceivable that the RNA-dependent RNA polymerase (RdRp) activity of NS5, a critical DENV protein, may serve as the target of EPMC. Once the virus enters host cells and commences replication, NS5 RdRp catalyzes the de novo synthesis of complementary negative-strand RNA from the positive-stranded viral RNA template^39^. Nonetheless, a comprehensive assessment of EPMC's impact on NS5 RdRp activity warrants further investigation. Additionally, it is noteworthy that the NS5 protein exhibits a high degree of conservation among all four DENV serotypes, characterized by a sequence identity ranging from 67 to 82%^39^. This conservation aligns with the observed anti-DENV effects of EPMC against all four DENV serotypes (Fig. 3), underlining the potential relevance of EPMC's mechanism of action to the broader spectrum of DENV variants. We conducted molecular docking simulations to predict the binding affinity of EPMC to the active sites of NS1, NS5 MTase, NS5 RdRp, NS2B-NS3 protease, or NS3 helicase. These sites have been previously implicated in the inhibition of DENV infection by other compounds, such as deoxycalyxin A^47^, S-adenosyl-l-homocysteine^48^, and compound 29^49^. Subsequently, we compared the binding affinities of EPMC with those of reference ligand to assess their efficacy. Unfortunately, none of the predicted dockings achieved a binding score higher than that of the reference ligand (Fig. S9). This outcome suggests limited interaction between EPMC and the tested viral protein targets. Despite the inability of molecular docking to identify specific viral protein targets for EPMC, our findings suggest the possibility that EPMC may instead target host proteins, thereby indirectly inhibiting viral infection. Nevertheless, further in vitro experiments are necessary to validate these findings and elucidate the underlying mechanism of action. For example, Brand et al. demonstrated that cyclolignan (S.71) reduces viral yield and interferes with DENV genome replication and/or polyprotein translation by altering microtubule distribution and inducing endoplasmic reticulum deterioration^40^. Jung et al. revealed that niclosamide inhibits viral RNA replication and also affects the maturation of DENV particles by inducing endosomal neutralization, resulting in the release of non-infectious immature virus particles^41^.

**Figure 8:**
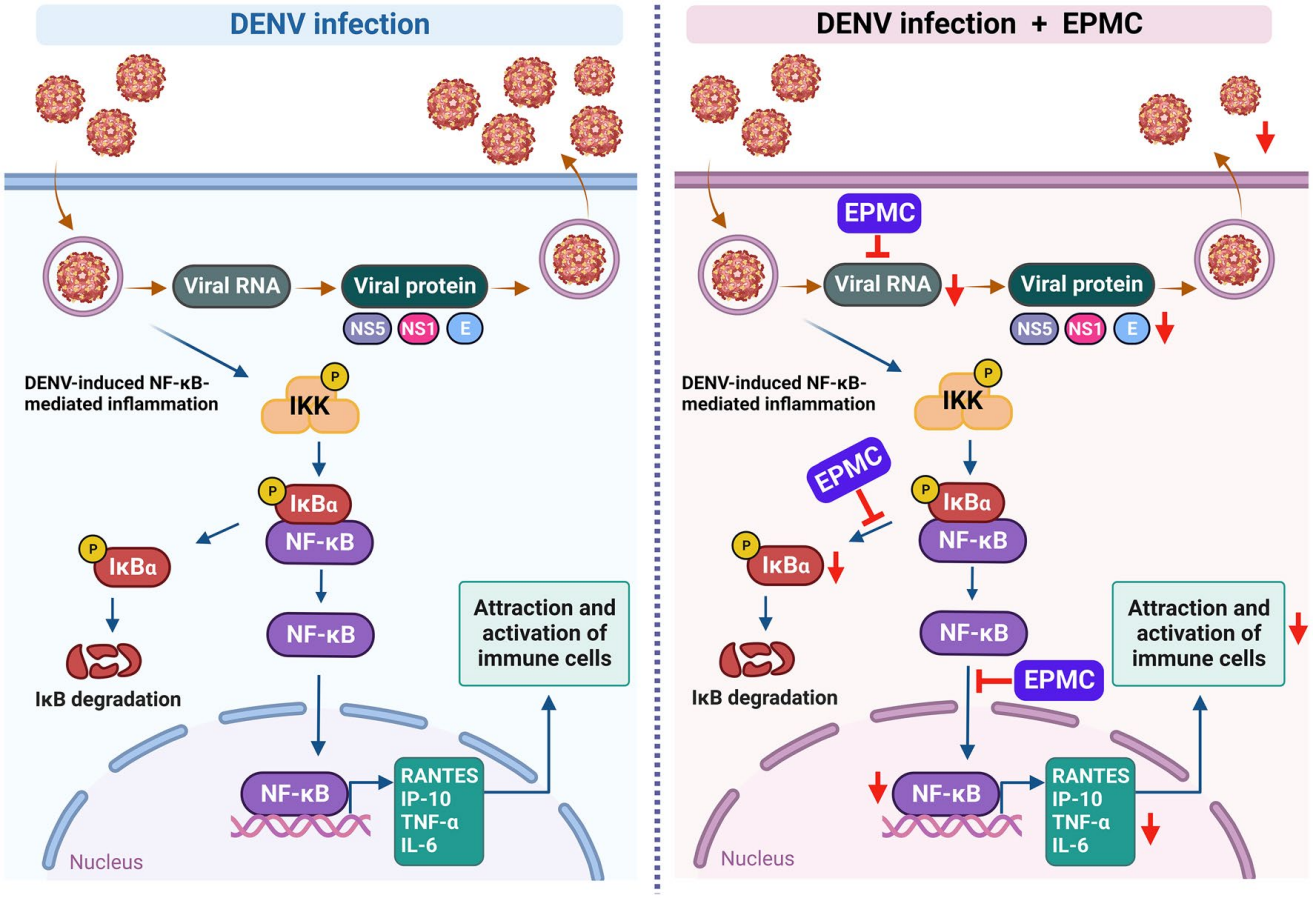
A proposed model for inhibiting DENV infection and suppressing DENV-induced cytokine/chemokine production by EPMC.

In addition to its pronounced antiviral efficacy, EPMC also exerts robust anti-inflammatory effects against DENV-2 infection by significantly attenuating both the mRNA and protein levels of DENV-2-induced pro-inflammatory cytokines and chemokines, including RANTES, IP-10, TNF-α, and IL-6 (Fig. 6). The overproduction of these inflammatory mediators is implicated in the pathogenic complications observed in severe dengue cases. The application of EPMC holds promise for potentially ameliorating these cytokine/chemokine surges, thereby contributing to the restoration of immunological homeostasis in DENV-infected patients. Our findings suggest that EPMC's anti-inflammatory activity may manifest both indirectly and directly. Indirectly, EPMC's anti-inflammatory potential may emanate from its ability to curtail viral replication and subsequent viral protein production. This is pivotal as DENV proteins, such as E protein, NS1, and NS5, are known to stimulate the release of host inflammatory cytokines through the activation of the NF-κB pathway^5,42,43^. Our results undeniably reveal that EPMC significantly reduces the levels of these DENV proteins, underscoring its potential in mitigating DENV-induced NF-κB-mediated inflammation (Figs. 2 and S2). Specifically, the translocation of NS5 into the nucleus has been associated with an elevation in RANTES expression through competitive binding with host protein Daxx. This interaction between NS5 and Daxx results in the liberation of NF-κB, thereby increasing its likelihood of binding to the RANTES promoter and subsequently enhancing RANTES expression^9,42^. Our findings are in consistent with previously documented results. In DENV-2-infected HepG2 cells, NS5 predominantly localizes within the nucleus, accompanied by the presence of nuclear NF-κB and heightened RANTES expression. Upon EPMC treatment in DENV-2-infected cells, a conspicuous reduction in nuclear NS5 and nuclear NF-κB levels is noted, concomitant with a significant suppression of RANTES expression. In addition to RANTES, the expression of IP-10, TNF-α, and IL-6, all of which are known to be regulated by NF-κB, is significantly diminished under EPMC treatment conditions (Figs. 6 and 7). Following EPMC treatment, the diminution of nuclear NS5 potentially preserves Daxx and NF-κB binding, leading to the inhibition of NF-κB-mediated cytokine and chemokine expression. To elucidate the molecular mechanisms of EPMC's action against DENV infection, we investigated its influence on the NF-κB signaling cascade. The results unequivocally indicate that EPMC significantly attenuates DENV-induced activation of phosphorylated IκB (P-IκB) and phosphorylated NF-κB p65 (P-NF-κB p65) (Fig. 7b,c). It can be inferred that the antiviral mechanism of EPMC involves the suppression of upregulated inflammatory cytokines induced by DENV through the inhibition of the NF-κB signaling pathway (Fig. 8).

Nonetheless, it is crucial to acknowledge the direct anti-inflammatory attributes of EPMC, which are underpinned by its immunomodulatory and anti-inflammatory properties, as evidenced in various inflammatory disease models beyond DENV infection. For instance, KG extracts have exhibited their anti-inflammatory and cytoprotective effects by targeting NF-κB activity^44^. Furthermore, EPMC has been identified as an anti-metastatic and chemosensitizing agent through its capacity to inhibit NF-κB activation in melanoma cells^45^. In a rat model of carrageenan-induced edema, which simulates an acute inflammatory condition, EPMC derived from KG extracts emerged as an active anti-inflammatory agent^24^. Similarly, the EPMC isolate from KG extract has been validated as an active constituent eliciting anti-inflammatory activity in both the carrageenan-induced granuloma air pouch model and the pleurisy model in rats^25^. Thus, it is plausible that EPMC treatment may aid in curtailing viral replication and managing cytokine storms associated with DENV infections by inhibiting the activation of the NF-κB signaling pathway.

Moreover, the SwissADME predicted that EPMC has good drug-likeness properties according to Lipinski’s, Egan’s, and Veber’s rules^29–31^. EPMC was predicted to be absorbed in the gastrointestinal tract and cross the blood–brain barrier. The ProTox-II classified EPMC as a non-toxic compound with LD_50_ values of 7900 mg/kg. The evidence indicating the nontoxicity of KG extract has been demonstrated in experimental toxicity studies in rodent models. Amuamuta et al. (2004) revealed a maximum single oral dose of KG extract up to 5000 mg/kg and a daily dose of 1000 mg/kg for 30 consecutive days in ICR mice^35^. Likewise, Kanjanapothi et al. reported that the maximum tolerated dose of KG extract in Sprague–Dawley rats via oral administration was up to 5000 mg/kg^46^. These findings highlight a promising pharmacokinetic profile for EPMC, coupled with favorable bioavailability and minimal toxicity properties, supporting its potential for further development as an anti-DENV drug. Subsequent stages in the development of EPMC as an antiviral agent necessitate comprehensive assessments encompassing efficacy, pharmacokinetics, toxicity, and safety in relevant animal models. The objective is to validate the compound's protective antiviral properties, low toxicity, reduced risk of viral resistance, and adequate stability for absorption and distribution.

In conclusion, our investigation has unveiled the remarkable antiviral potential of the *K. galanga* L. (KG) extract against DENV-2, marking a novel contribution to the field of dengue therapeutics. EPMC, identified as the principal bioactive compound within *K. galanga* L., has exhibited robust antiviral activity encompassing all four dengue serotypes. EPMC treatment demonstrated a striking reduction in the population of infected cells, along with a notable decrease in virus production and the synthesis of viral proteins. The anti-DENV activity of EPMC was attributed to its interference with both the early and late stages of the DENV life cycle, effectively impeding intracellular replication. Crucially, EPMC exhibited a dual role in mitigating dengue pathology. Beyond its antiviral prowess, it significantly curtailed cytokine and chemokine production by virtue of its capability to inhibit NF-κB nuclear translocation and deactivate the NF‑κB signaling pathway. This pathway holds paramount importance in orchestrating the inflammatory response during DENV infection. In light of these compelling findings, both *K. galanga* L. and its major bioactive constituent, EPMC, emerge as promising and multifaceted candidates for the treatment of dengue, warranting further exploration in the quest to combat this challenging viral affliction.

## Materials and methods

### Cell cultures and DENV propagation

Human hepatocellular carcinoma (HepG2; ATCC HB-8065), human lung epithelial carcinoma (A549; ATCC CCL-185), and African green monkey kidney (Vero; ATCC CCL-81) cells were meticulously cultivated in accordance with their specific growth and maintenance requirements, following well-established protocols from prior studies^17,18^. DENV-1 strain Hawaii, DENV-2 strain 16681, DENV-3 strain H87, and DENV-4 strain H241 were propagated in *Aedes albopictus* C6/36 cells, underwent rigorous titration through the FFU assay, and were then stored at − 80 °C until their intended use^18^.

### *Kaempferia galanga* L. extraction

The dried and powdered rhizomes of *K. galanga* L. were sourced from Thaprachan herbs Co., Ltd. in Bangkok, Thailand. The plant collection and use were in accordance with all the relevant guidelines. Authentication of plant materials was conducted at the herbarium of the Department of Forestry in Bangkok, Thailand, where the herbarium vouchers are stored. To obtain the crude extract of *K. galanga* L., a sonication method was employed. In brief, 100 g of powdered plant rhizome were immersed in 200 ml of absolute ethanol for 30 min, maintaining a 1:2 w/v ratio. This process was iterated five times to ensure thorough extraction. The resulting extract was meticulously filtered using Whatman No. 1 filter paper and subsequently evaporated at 50 °C under reduced pressure through a rotary evaporator. The final extract yield amounted to 4.38% and was subsequently subjected to freeze-drying, after which it was stored at − 20 °C until needed for further experimentation.

### High performance liquid chromatography (HPLC)

The analysis of the KG extract was performed using a state-of-the-art HPLC 1260 Series system (Agilent Technologies, CA, USA). To accurately detect the presence of ethyl-p-methoxycinnamate (EPMC) within the KG extract, a highly pure EPMC compound sourced from TCI in Chuo-ku, Tokyo, Japan, was employed as a reference marker. The separation process involved the use of a Hypersil BDS Column (100 × 4.6 mm i.d., 3.5 μm particle size), a reversed phase C_18_ column (Thermoscientific, MA, USA), coupled with a C_18_ guard column. Elution was achieved through a precise isocratic solvent system, consisting of a mixture of 0.5% acetic acid in water and methanol (40:60, v/v). The flow rate was consistently maintained at a constant 1.0 ml/min, with temperature control set precisely at 25 °C. The detection of compounds was accomplished using a diode array detector, which was specifically configured to a wavelength of 314 nm. A 10 μl injection volume was utilized for the analysis. By comparing the bioactive compound profiles in the KG extract with the well-defined standard EPMC reference, a comprehensive and accurate analysis was conducted, allowing for the identification of constituents within the extract.

### Cytotoxicity assay

To evaluate the cytotoxicity of both the KG extract and EPMC, we employed the PrestoBlue Cell Viability Reagent (Invitrogen, Carlsbad, CA, USA). HepG2 or A549 cells were initially seeded in 96-well plates, with each well containing 2.5 × 10^4^ cells, and allowed to incubate overnight. Subsequently, following 48 h of culture with either the KG extract or EPMC, we introduced PrestoBlue to the cells. To measure cell viability, we recorded the absorbance at 570/600 nm using the Synergy H1 microplate reader (Biotek Instruments, Winooski, VT, USA). The analysis of the maximum non-toxic dose (MNTD_80_) and the half-maximal cytotoxic concentration (CC_50_) was carried out using CalcuSyn v2.11 software (Biosoft, Cambridge, UK). The MNTD_80_ represents the dose at which 80% of the cells maintain viability, while the CC_50_ signifies the concentration at which 50% cytotoxicity becomes evident. This comprehensive assessment allowed us to gain valuable insights into the impact of the KG extract and EPMC on cell viability.

### DENV infection and compound treatment

To assess the anti-DENV activity of both the KG extract and EPMC in HepG2 or A549 cells, an overnight cell culture was first established, followed by infection with DENV-2 at a multiplicity of infection (MOI) of 5. After a 2-h incubation at 37 °C to facilitate virus absorption, unbound DENV was meticulously removed, and the cells were washed with PBS. Subsequently, the cells were exposed to culture medium containing either the KG extract, EPMC (TCI, Oxford, UK), ribavirin (RV) (Sigma-Aldrich, Saint Louis, MO, USA), or the respective solvent. At 24 hpi, the treated cells were examined for DENV-infected cells using flow cytometry. Additionally, culture supernatants were collected to determine virus production via the FFU assay. For determination of EC_50_s, DENV-2-infected cells were treated with EPMC at concentrations of 0, 12.5, 25, 50, 75, 100, 125, 200, 250, 400, and 500 μM. At 24 hpi, the progeny virus production in the culture supernatants was determined using the FFU assay. The EC_50_ of EPMC was determined based on its effect on progeny virus production using CalcuSyn v2.11 software.

To evaluate the antiviral activity of EPMC across all four DENV serotypes, HepG2 cells were cultured overnight and subsequently infected with DENV-1, DENV-2, DENV-3, and DENV-4 at MOI 5, 5, 10, and 5, respectively. Following infection, cells were exposed to EPMC treatment or left untreated for a duration of 24 h. Post-treatment, culture supernatants were harvested to quantify virus production via the FFU assay. This approach allowed for the comprehensive assessment of EPMC's antiviral efficacy against all four DENV serotypes.

### Focus forming unit (FFU) assay

In order to evaluate the impact of the KG extract and EPMC on DENV replication, we employed a well-established FFU assay, similar to the approach detailed in a prior study^18^. This assay allowed us to quantitatively measure the levels of newly synthesized virus within the culture supernatant using Vero cells as the host system.

### Flow cytometry

To enumerate the DENV-infected cells, we initiated the process by gently detaching the cells using a solution composed of 0.1% trypsin and 2.5 mM EDTA in PBS. Subsequently, the cells were fixed with 4% paraformaldehyde in PBS and made permeable with 0.2% Triton X-100 in PBS. To identify DENV-infected cells, we utilized the mouse monoclonal anti-DENV-E antibody (4G2), followed by staining with Alexa Fluor 488-conjugated goat anti-mouse IgG (Molecular Probe, Eugene, OR, USA). The enumeration of these DENV-infected cells was precisely analyzed through the use of a BD Accuri C6 Flow Cytometer (BD Biosciences, Franklin Lakes, NJ, USA).

### Time-of-drug-addition assay

To elucidate the role of EPMC in the DENV life cycle, we initiated experiments using HepG2 cells seeded in 12-well plates at a density of 3.5 × 10^5^ cells per well. EPMC was introduced at various concentrations under three distinct conditions: preinfection, coinfection, and postinfection scenarios. In the preinfection condition, cells were incubated with EPMC (ranging from 0 to 500 µM) for a duration of 2 h. Subsequently, the cells were thoroughly washed prior to infection with DENV-2 at a MOI of 5. For the coinfection condition, cells were simultaneously infected with DENV-2 and treated with EPMC for a 2-h period. After this incubation, both excess viruses and EPMC were meticulously removed through a PBS washing step, and fresh medium was introduced. Under the postinfection condition, EPMC was introduced at 2 hpi and was maintained throughout the course of the experiment. At the 24 hpi time point, the cells were collected for the detection of DENV-infected cells using flow cytometry.

To pinpoint the specific inhibitory stage of EPMC during the postinfection phase, HepG2 cells were infected with DENV-2 for 2 h. Unbound virus was removed by washing with PBS, then 500 μM EPMC was added at 2, 4, 6, 8, 10, 12, and 14 hpi and maintained throughout the infection. EPMC was also added simultaneously with DENV-2 infection for 2 h (0–2 h), after which the media was removed, the cells were washed, and replaced with fresh media to observe the effect of EPMC during DENV infection. At 24 hpi, the culture supernatants were collected for virus titration by FFU assay and the cells were harvested for quantitation of intracellular viral RNA copy number by qRT-PCR. This comprehensive approach allowed us to gain valuable insights into the precise mechanisms and stages at which EPMC exerts its inhibitory effects within the DENV life cycle.

### Quantitative real‑time reverse transcription polymerase chain reaction (qRT‑PCR) analysis

To quantify the levels of DENV RNA, we initiated the process by extracting total RNA from both mock and DENV-infected cells, with or without treatment, using the highly effective Trizol reagent (Invitrogen). Subsequently, cDNA synthesis was performed using AMV Reverse Transcriptase (Promega, Madison, WI, USA). The amplification step was carried out with SYBR Green master mixes (Bio-Rad, Hercules, CA, USA) in the presence of cDNA templates and specific primers designed for DENV NS1. To facilitate accurate quantification of DENV RNA levels, we utilized DENV RNA standards with known copy numbers per microliter, creating a standard curve that served as a reference^18^.

For the assessment of cytokine and chemokine transcription, a parallel approach was adopted. Total RNA was extracted, and cDNA synthesis was executed using Superscript III reverse transcriptase (Invitrogen). Real-time PCR amplification of cDNA was carried out employing a reaction mixture of SYBR Green master mixes and specific primers. The real-time PCR conditions closely followed the protocol outlined in a previous study^18^. The relative mRNA expression was normalized against the β-actin mRNA level using the comparative Ct method. Fold changes in mRNA expression were calculated for cells infected with DENV-2 in the presence or absence of EPMC, relative to mock-infected cells. The fold change value was expressed as a percentage of transcription. The data are presented as the percentage of transcription relative to that of the untreated DENV-2 infected cells, which was defined as 100%.

### Multiplexed bead-based immunoassays

To assess the impact of EPMC treatment on the secretion of cytokines and chemokines from DENV-infected HepG2 cells, we gathered culture supernatants at the 24 hpi time point. The levels of specific cytokines and chemokines, namely RANTES, IP-10, TNF-α, and IL-6, were quantitatively analyzed. This analysis was conducted using the BD Accuri C6 Flow Cytometer in conjunction with the BD CBA Human Soluble Protein Flex Set system (BD Biosciences).

### Immunoblot analysis

To evaluate the impact of EPMC treatment on DENV-2 protein production, DENV-infected cells were subjected to lysis using RIPA buffer. Equal quantities of proteins were subsequently separated through SDS-PAGE and transferred onto nitrocellulose membranes. Detection of DENV NS5, NS1, and E proteins was carried out employing specific antibodies: mouse anti-DENV-NS5 (Invitrogen), anti-DENV-NS1 (DN3) (Abcam, Cambridge, UK), and anti-DENV-E (4G2). Subsequently, these were probed with HRP-conjugated rabbit anti-mouse IgG antibodies (DAKO) and visualized through an enhanced chemiluminescence system (Perkin Elmer, Waltham, MA, USA).

To investigate the effect of EPMC treatment on NF-κB activation, the subcellular fractionation of cytoplasmic and nuclear proteins was executed using the Subcellular Protein Fractionation Kit (Thermo Scientific, Rockford, IL, USA). The expression levels of NF-κB p65, phosphorylated NF-κB p65 (P-NF-κB p65), IκB, and phosphorylated IκB (P-IκB) were determined through immunoblot analysis. Specifically, we employed rabbit anti-NF-κB p65, anti-P-NF-κB p65, anti-IκB, and anti-P-IκB antibodies (Cell Signaling Technology, Danvers, MA, USA). To ensure data integrity, GAPDH protein was detected using an anti-GAPDH antibody (Santa Cruz Biotechnology, Dallas, TX, USA), serving as an internal control. Additionally, lamin A was detected using a mouse monoclonal anti-lamin A antibody (Cell Signaling Technology) as a nuclear fraction marker. Subsequent to protein band detection, quantification was performed utilizing ImageJ 1.52v software (https://imagej.nih.gov/ij).

### Indirect immunofluorescence assay

To assess the impact of KG extract or EPMC on DENV infection, cells cultured on glass coverslips underwent a series of steps. First, they were fixed with a 4% paraformaldehyde solution in PBS, followed by permeabilization using 0.2% Triton X-100 in PBS. Subsequently, the cells were incubated with the 4G2 antibody. After thorough washing, a mixture containing Cy3-conjugated goat anti-mouse IgG (Invitrogen) was applied, along with Hoechst 33342 for nuclear staining (Invitrogen). The resulting fluorescence images were visualized using a laser scanning confocal microscope (LSM 800, Zeiss, Jena, Germany).

To investigate the influence of EPMC on the presence of DENV-2 NS5 and the nuclear translocation of NF-κB p65, the cells were subjected to a similar procedure. They were incubated with a mixture comprising mouse anti-DENV-NS5 and rabbit anti-NF-κB p65 antibodies, followed by a combination of Cy3-conjugated goat anti-mouse IgG, Alexa Fluor 488-conjugated donkey anti-rabbit IgG, and Hoechst 33342^18^.

### Prediction of drug-like properties and ADMET

The drug-like properties of EPMC were determined using the Swiss ADME webserver (http://www.swissadme.ch, accessed on 04 January 2024)^28^. The following parameters like molecular weight (MW), topological polar surface area (TPSA), number of hydrogen bond acceptors (nOHNH), number of hydrogen bond donors (nON), water partition coefficient (WLOGP), and a number of rotatable bonds (nrotb) were computed to incorporate for drug-like properties prediction based on Lipinski’s rule of 5^29^, Egan’s rule^30^, and Veber’s rule^31^.

ProTox II webserver (http://tox.charite.de/protox_II, accessed on 04 January 2024), a virtual laboratory experiment designed for predicting the toxicities of small molecules^33^, was applied to analyze toxicity properties of EPMC. The analysis considered parameters such as acute oral toxicity, hepatotoxicity, cytotoxicity, carcinogenicity, mutagenicity, and immunotoxicity.

### Molecular docking

Molecular docking was performed to elucidate the ability of EPMC to target DENV proteins. Five crystal structures of three DENV proteins including NS1 (PDB entry 4O6B)^47^, NS5 (PDB entry 1R6A and 5JJR)^48,49^, and NS3 (PDB entry 4M9K and 2BHR)^50,51^ were extracted from RCSB protein data bank (https://www.rcsb.org/ accessed on 15 January 2024). The MOL2 file of EPMC was derived from MolView web-based program (https://molview.org/ accessed on 15 January 2024). Molecular docking was conducted by using GOLD Protein–ligand docking software followed the default setting^52^. The reference ligands were extracted from the structure and those structures were defined as the receptors for docking process. The EPMC or reference ligands were docked to the protein receptors. The fitness score for each protein–ligand interaction, either reference ligands or EPMC, was obtained from ChemPLP (ChemPLP score). The achieved fitness scores were ranked, and the highest scores were compared between EPMC and reference ligand. Furthermore, the detailed intermolecular interaction was analyzed using View Interaction mode of Discovery Studio Visualizer^53^ (BIOVIA, Dassault Systèmes, BIOVIA Discovery Studio Visualizer, San Diego: Dassault Systèmes, 2024, https://discover.3ds.com/discovery-studio-visualizer-download). The figures of docking poses with high scored and 2D interaction were prepared using Discovery Studio Visualizer.

### Statistical analysis

Statistical analyses were performed utilizing Graph-Pad Prism v5.01 software (GraphPad Software, Inc., www.graphpad.com). Each experiment was carried out independently three times, and the data are presented as the mean ± standard deviation (SD). To assess statistical variances in means, we employed either an unpaired Student's t-test or a one-way ANOVA test, followed by Tukey's post hoc test for multiple comparisons. Significance levels were represented as follows: *p < 0.05, **p < 0.01, ***p < 0.001 when compared with untreated DENV-infected cells.

### Supplementary Information

Supplementary Information.

## Data Availability

All data generated or analyzed during this study are included in this published article and its supplementary information file.
