# Supplementary material for: Dual action effects of ethyl-p-methoxycinnamate against dengue virus infection and inflammation via NF-κB pathway suppression
Source: Sci Rep. 2024 Apr 23;14:9322. doi: 10.1038/s41598-024-60070-1 (PMC11039621; doi:10.1038/s41598-024-60070-1)
Supplement: Supplementary file 1 — Supplementary Information. [file 41598_2024_60070_MOESM1_ESM.pdf]

Dual action effects of ethyl-p-methoxycinnamate against dengue virus infection and inflammation via NF-κB pathway suppression

Mayuri Tarasuk[1a], Pucharee Songprakhon[2a], Phunuch Muhamad[3], Aussara Panya[4], Pachara Sattayawat[4], Pa-thai Yenchitsomanus[2*]

[1] *Graduate Program in Bioclinical Sciences, Chulabhorn International College of Medicine, Thammasat University, Pathum Thani, Thailand*

[2] *Division of Molecular Medicine, Research Department, Faculty of Medicine Siriraj Hospital, Mahidol University, Bangkok, Thailand*

[3] *Drug Discovery and Development Center, Office of Advanced Science and Technology, Thammasat University, Pathum Thani, Thailand*

[4] *Cell Engineering for Cancer Therapy Research Group, Faculty of Science, Chiang Mai University, Chiang Mai, Thailand*

[a] *These authors contributed equally*

Correspondence to:

Pa-thai Yenchitsomanus, PhD

Professor of Molecular Genetics and Medical Molecular Biology

Siriraj Center of Research Excellence for Cancer Immunotherapy (SiCORE-CIT), and

Division of Molecular Medicine, Research Department,

Faculty of Medicine Siriraj Hospital, Mahidol University

2 Wanglang Road, Bangkoknoi, Bangkok 10700, Thailand

Tel: (+66) 2-419-2777; Fax: (+66) 2-411-0169

E-mail: pathai.yen@mahidol.ac.th, ptyench@gmail.com

Supplementary Information

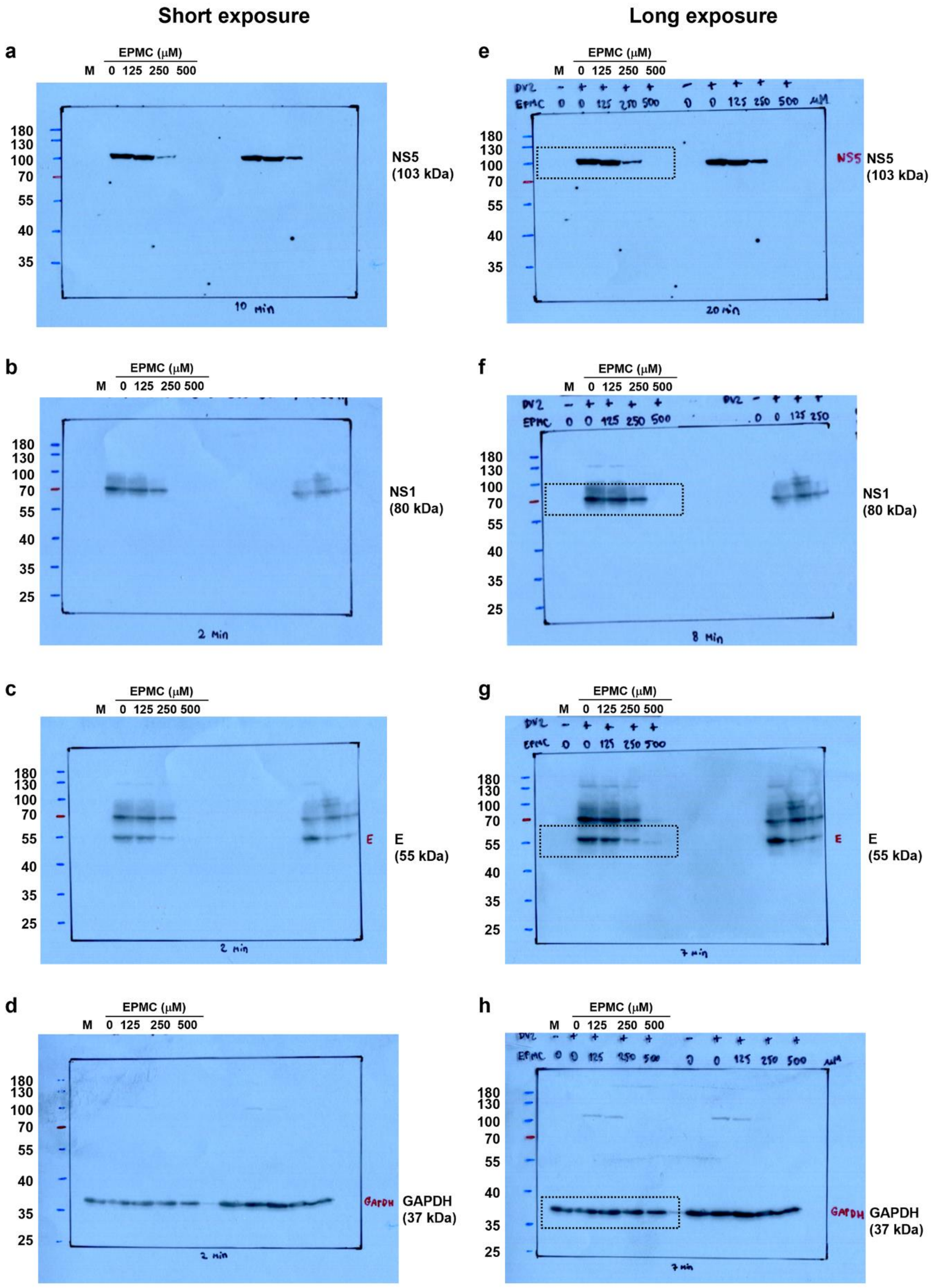

Supplementary Figure S1. Original and full-length blots of Figure 2d. HepG2 cells were infected with DENV-2 and treated with EPMC at 2 hpi. Cells were harvested at 24 hpi for protein extraction. Immunoblots shown in the main figure are cropped images as indicated by dashed lines. The results are the representatives from three independent experiments. All blots were derived from the same experiment and were processed in parallel. Each membrane was individually incubated with a specific antibody to detect either DENV NS5 (a, e), NS1 (b, f), or E proteins (c, g). Subsequently, the same membrane was incubated with anti-GAPDH antibody to detect GAPDH as an internal control (d, h). The membranes were exposed to X-ray films for multiple exposure times, whether short or long, as indicated.

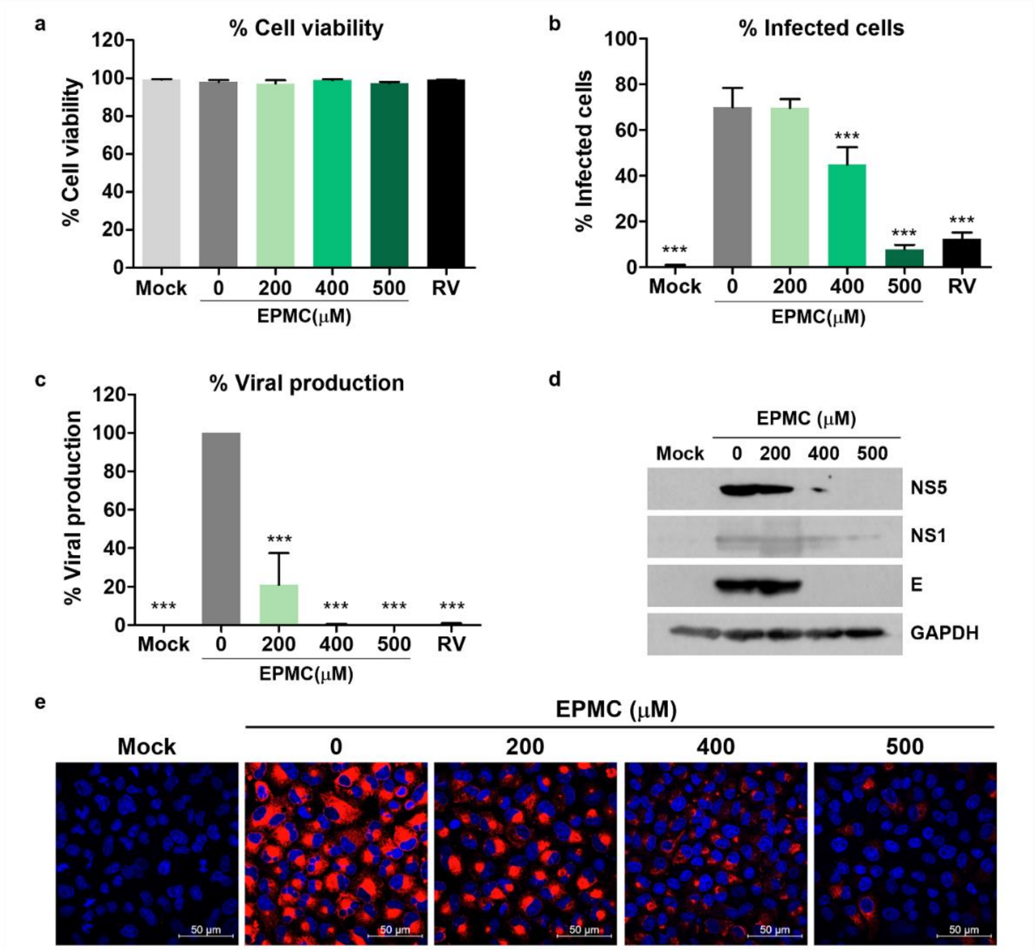

Supplementary Figure S2. EPMC attenuates DENV-2 infection, progeny virus production, and protein expression in A549 cells. A549 cells were infected with DENV-2 (MOI of 5) and treated with either EPMC (0-500 μM) or RV (100 μM) for 24 h. (a) Assessment of cell viability, expressed as percentages, through the trypan blue exclusion assay. (b) Determination of the proportion of infected cells via flow cytometry. (c) Quantification of progeny virus production in culture supernatants using the FFU assay. The results are presented as mean ± SD from three independent experiments. Statistical differences, compared to the control group, were calculated using one-way ANOVA and Tukey's HSD test (***$p < 0.001$). (d) Immunoblot analysis revealing DENV-2 NS5, NS1, and E protein levels in lysates of infected cells. The original blots are presented in Supplementary Figure S3. (e) Visualization of the DENV-2 E protein (in red) through immunofluorescence assay, accompanied by nuclear staining using Hoechst (in blue).

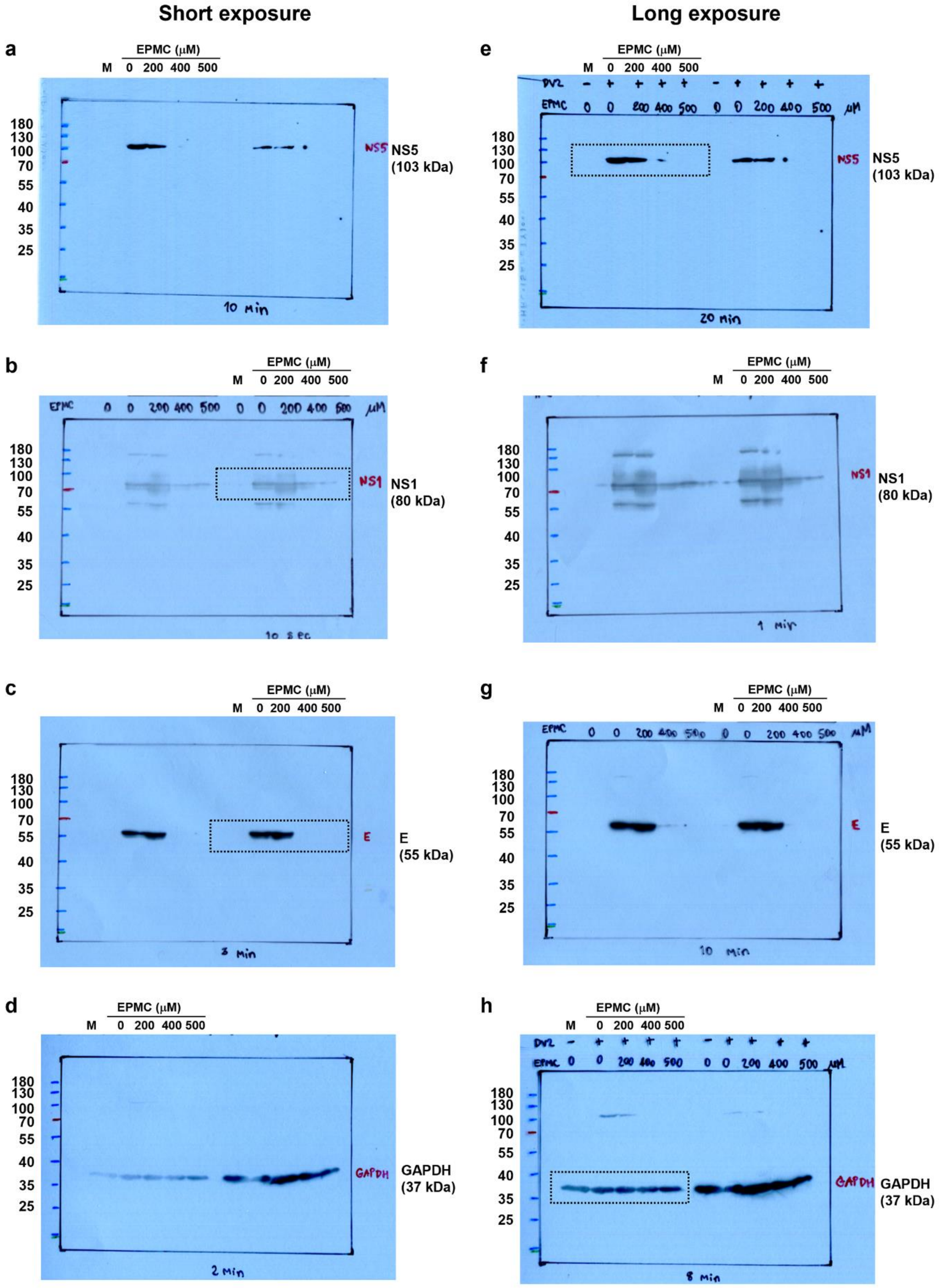
Short exposure
Long exposure
a
EPMC (μM)
M 0 200 400 500
180
130
100
70
55
40
35
25
NS5
(103 kDa)
10 min
e
EPMC (μM)
M 0 200 400 500
NS5
(103 kDa)
20 min
b
EPMC (μM)
M 0 200 400 500
NS1
(80 kDa)
f
EPMC (μM)
M 0 200 400 500
NS1
(80 kDa)
1 min
c
EPMC (μM)
M 0 200 400 500
E
(55 kDa)
3 min
g
EPMC (μM)
M 0 200 400 500
E
(55 kDa)
10 min
d
EPMC (μM)
M 0 200 400 500
GAPDH
(37 kDa)
2 min
h
EPMC (μM)
M 0 200 400 500
GAPDH
(37 kDa)
8 min

Supplementary Figure S3. Original and full-length blots of Supplementary Figure S2d. A5492 cells were infected with DENV-2 and treated with EPMC at 2 hpi. Cells were harvested at 24 hpi for protein extraction. Immunoblots shown in the main figure are cropped images as indicated by dashed lines. The results are the representatives from three independent experiments. All blots were derived from the same experiment and were processed in parallel. Each membrane was individually incubated with a specific antibody to detect either DENV NS5 (a, e), NS1 (b, f), or E proteins (c, g). Subsequently, the same membrane was incubated with anti-GAPDH antibody to detect GAPDH as an internal control (d, h). The membranes were exposed to X-ray films for multiple exposure times, whether short or long, as indicated.

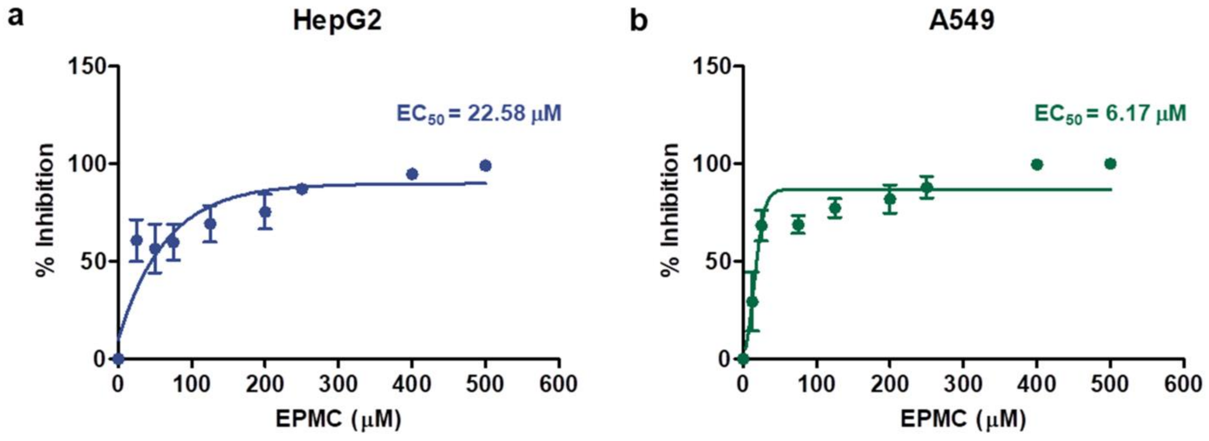

Supplementary Figure S4. Dose response curve analysis of EPMC against DENV-2. HepG2 (a) and A549 (b) cells were infected with DENV-2 at MOI 5 and treated with 0-500 μM of EPMC for 24 h. The culture supernatants were collected for the FFU assay. Dose-response curves show the inhibition of viral production at increasing concentrations of EPMC in relation to the untreated control. EC_{50} values were determined using CalcuSyn™ v2.11 software. The data are presented as mean ± SD from three independent experiments.

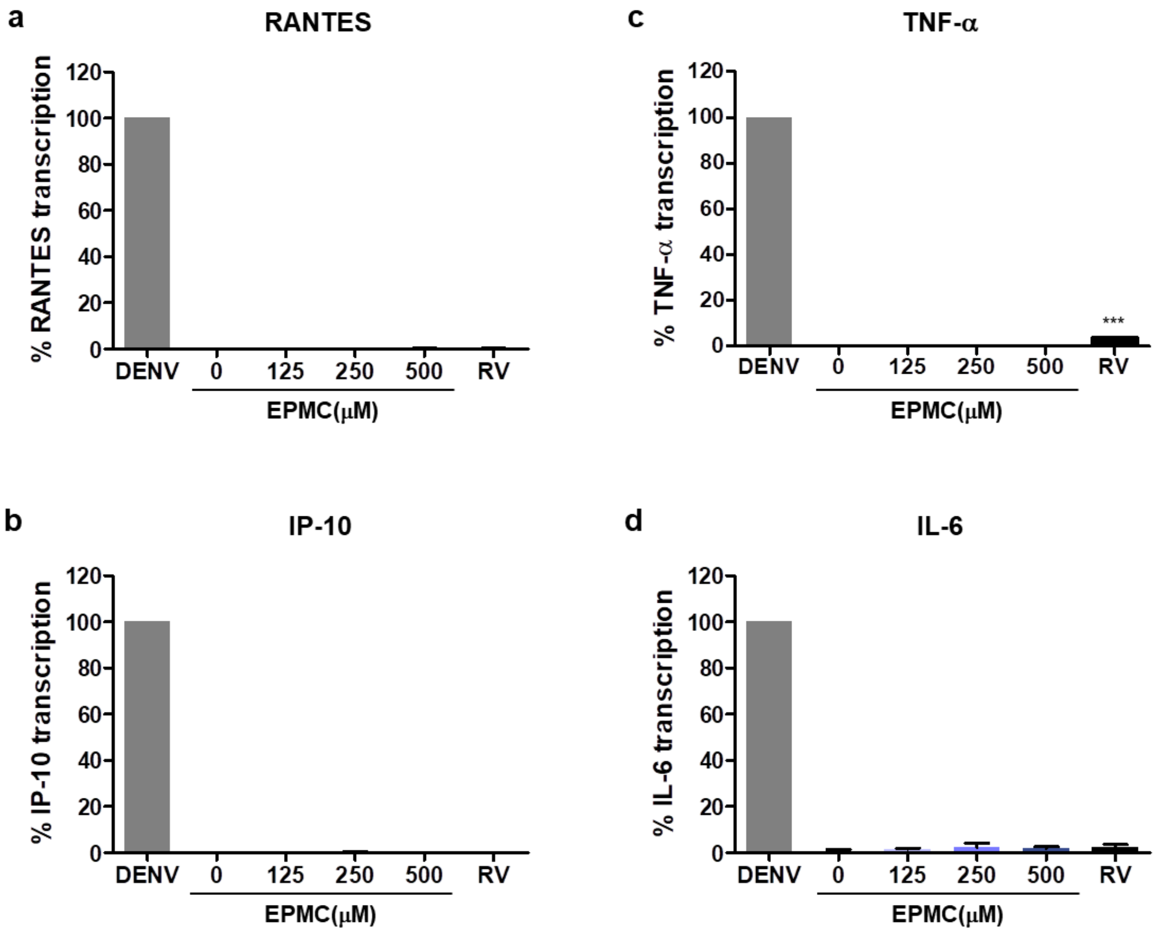

Supplementary Figure S5. Effect of EPMC on cytokine/chemokine expression in mock-infected HepG2 cells. HepG2 cells were cultured overnight and subsequently treated with either EPMC (0-500 μM) or RV (100 μM) for 24 h. HepG2 cells were infected with DENV-2 (MOI of 5) and used as a positive control for DENV-2-induced cytokine/chemokine expression. (a-d) The mRNA levels of RANTES, IP-10, TNF-α, and IL-6 in mock-infected HepG2 cells were determined by qRT-PCR. The transcription level of each cytokine/chemokine in untreated DENV-infected cells was defined as 100%. Statistical differences, compared to the untreated mock-infected cells, were calculated using one-way ANOVA and Tukey's HSD test (***$p < 0.001$).

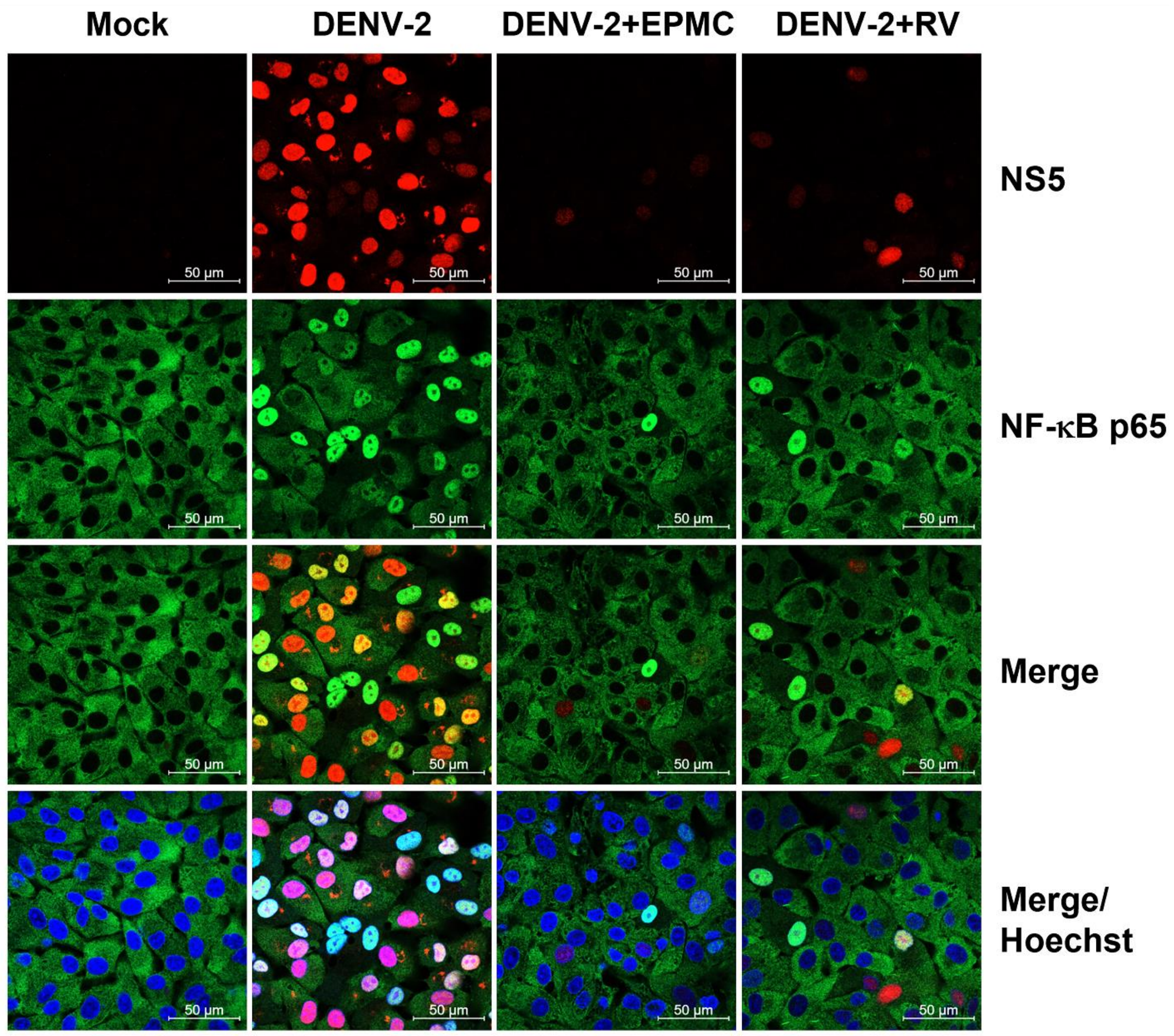

Supplementary Figure S6. EPMC inhibits NF-κB nuclear translocation in DENV-2-infected A549 cells. A549 cells were infected with DENV-2 (MOI of 5) and treated with either EPMC (500 μM) or RV (100 μM) for 24 h. The immunofluorescence assay illustrates the expression and intracellular localization of DENV NS5 (in red) and NF-κB p65 (in green). Nuclear staining is accomplished using Hoechst dye (in blue).

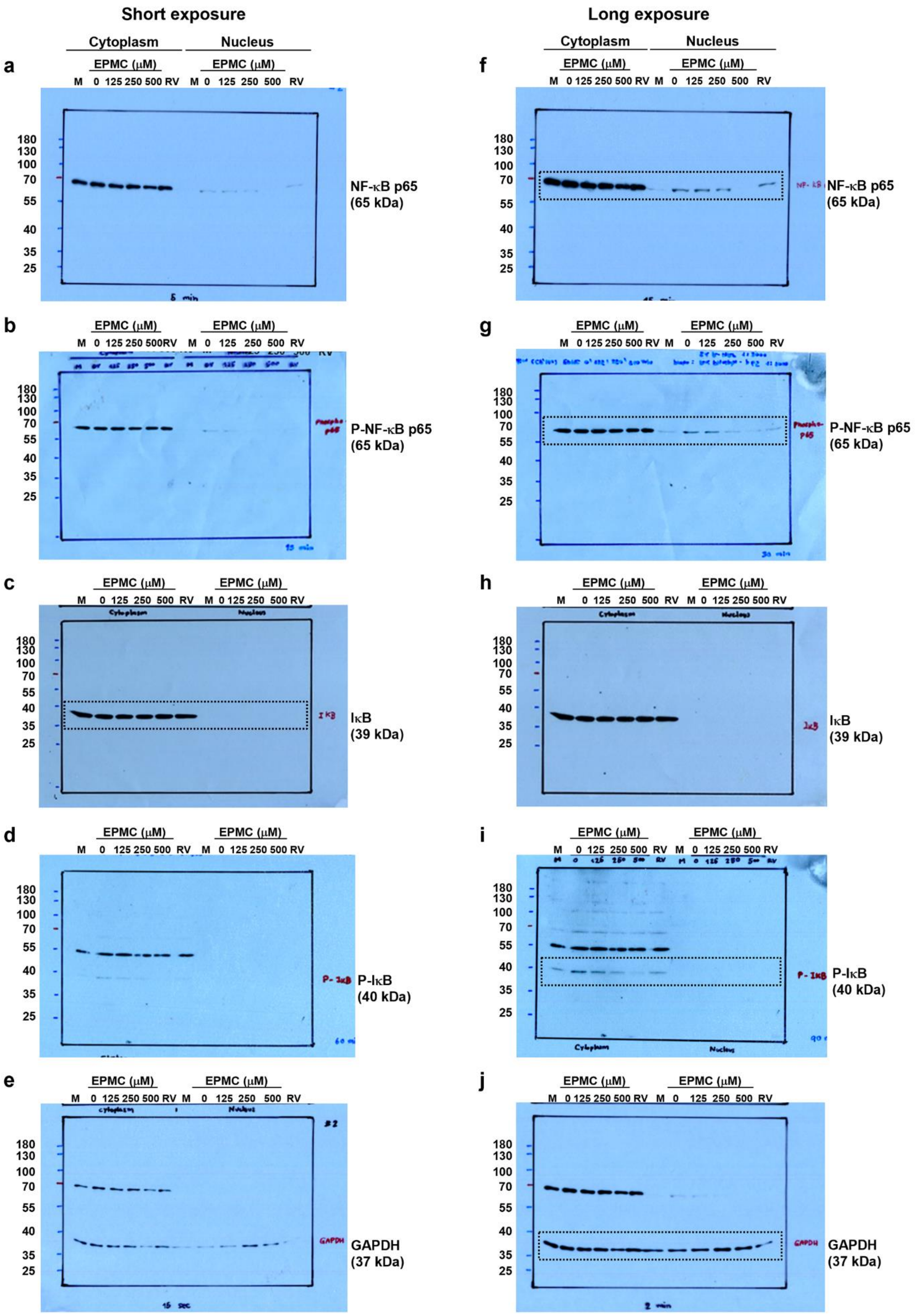
Short exposure
Long exposure
Cytoplasm
Nucleus
a
b
c
d
e
f
g
h
i
j
EPMC (μM)
M 0 125 250 500 RV M 0 125 250 500 RV
180
130
100
70
55
40
35
25
NF-κB p65 (65 kDa)
P-NF-κB p65 (65 kDa)
IκB (39 kDa)
P-IκB (40 kDa)
GAPDH (37 kDa)

Supplementary Figure S7. Original and full-length blots of Figure 7b. HepG2 cells were infected with DENV-2 and treated with either EPMC or RV at 2 hpi. Cells were harvested at 24 hpi for cytoplasmic and nuclear protein extraction. Immunoblots shown in the main figure are cropped images as indicated by dashed lines. The results are the representatives from three independent experiments. All blots were derived from the same experiment and were processed in parallel. Each membrane was individually incubated with a specific antibody to detect either NF-κB p65 (a, f), IκB (b, g), P-NF-κB p65 (c, h), or P-IκB (d, i). Subsequently, the same membrane was incubated with anti-GAPDH antibody to detect GAPDH as an internal control (e, j). The membranes were exposed to X-ray films for multiple exposure times, whether short or long, as indicated.

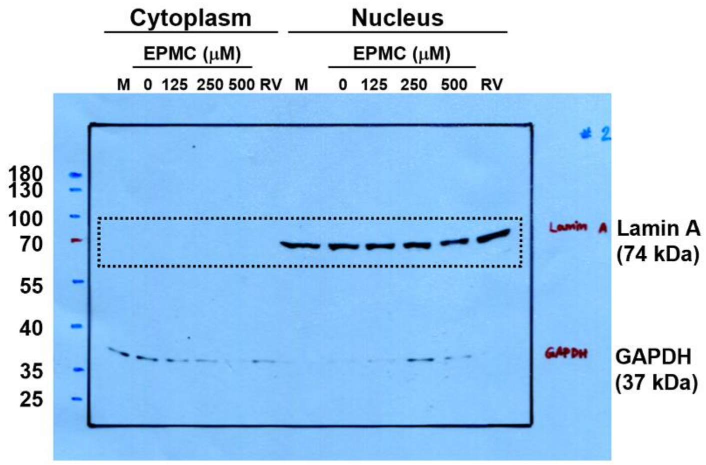

Supplementary Figure S8. Original and full-length blot of Figure 7b for lamin A protein detection. HepG2 cells were infected with DENV-2 and treated with either EPMC or RV at 2 hpi. Cells were harvested at 24 hpi for cytoplasmic and nuclear protein extraction. The membrane was incubated with anti-lamin A antibody to detect lamin A protein as a nuclear fraction marker. The immunoblot shown in the main figure is a cropped image, as indicated by dashed lines. The membrane was exposed to X-ray film for a single exposure time.

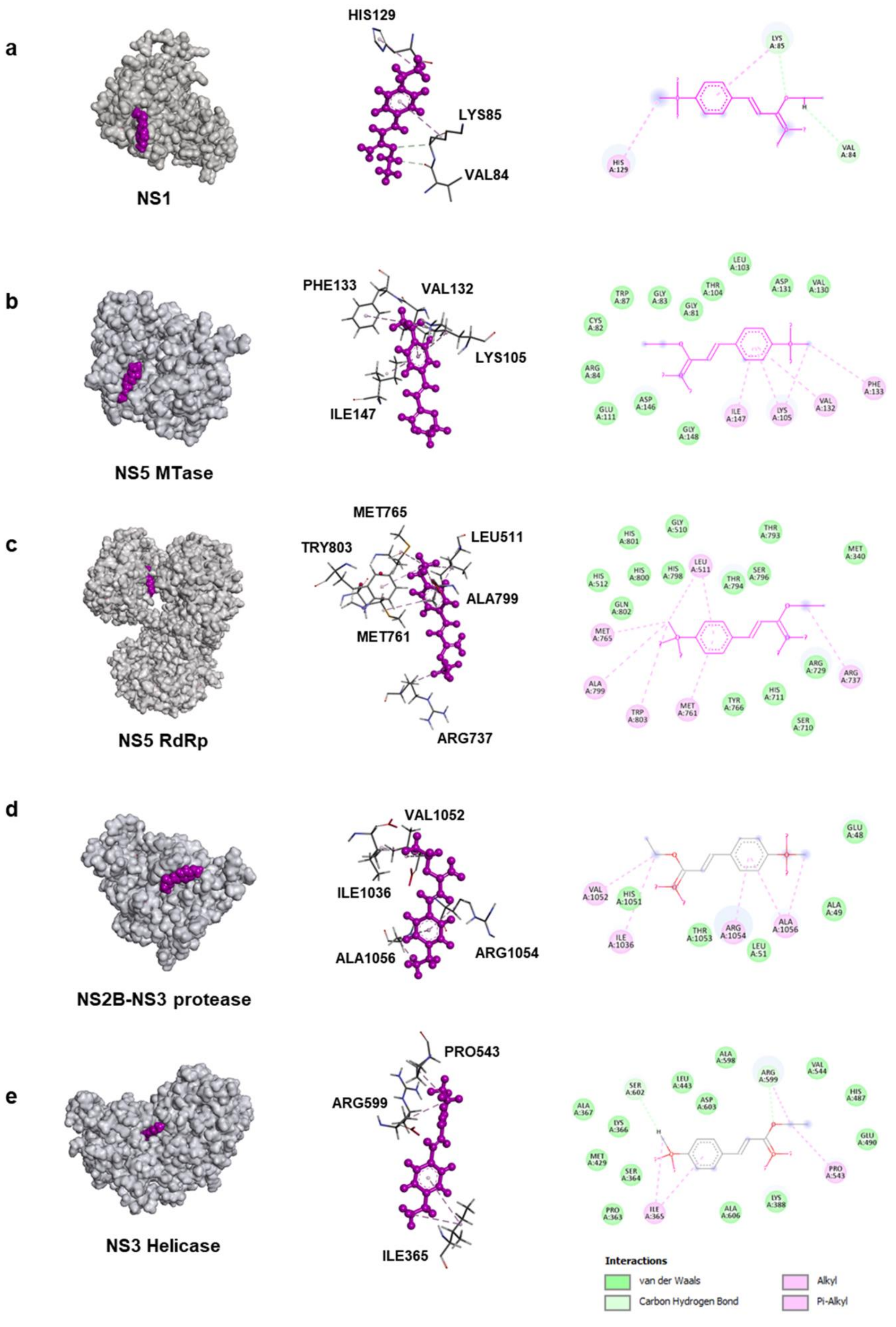
a
NS1
HIS129
LYS85
VAL84
LYS A:85
HIS A:129
VAL A:84
b
NS5 MTase
PHE133
VAL132
LYS105
ILE147
LEU A:103
TRP A:87
GLY A:83
THR A:104
GLY A:81
ASP A:131
VAL A:130
CYS A:82
ARG A:84
GLU A:111
ASP A:146
GLY A:148
ILE A:147
LYS A:105
VAL A:132
PHE A:133
c
NS5 RdRp
MET765
TRY803
LEU511
ALA799
MET761
ARG737
HIS A:801
GLY A:510
THR A:793
MET A:340
HIS A:512
HIS A:800
HIS A:798
LEU A:511
THR A:794
SER A:796
GLN A:802
MET A:765
ARG A:729
ARG A:737
ALA A:799
TRP A:803
MET A:761
TYR A:766
HIS A:711
SER A:710
d
NS2B-NS3 protease
VAL1052
ILE1036
ALA1056
ARG1054
GLU A:48
VAL A:1052
HIS A:1051
ILE A:1036
THR A:1053
ARG A:1054
ALA A:1056
LEU A:51
ALA A:49
e
NS3 Helicase
PRO543
ARG599
ILE365
ALA A:598
VAL A:544
SER A:602
LEU A:443
ARG A:599
HIS A:487
ALA A:367
ASP A:603
LYS A:366
GLU A:490
MET A:429
SER A:364
PRO A:543
PRO A:363
ILE A:365
ALA A:606
LYS A:388
Interactions
van der Waals
Carbon Hydrogen Bond
Alkyl
Pi-Alkyl

Supplementary Figure S9. Molecular interactions of EPMC with DENV non-structural protein targets. The interaction of EPMC (in purple stick model) with (a) NS1, (b) NS5 MTase domain, (c) NS5 RdRP domain, (d) NS2-NS3 protease domain, and (e) NS3 helicase domain were predicted (left and middle). Intermolecular interaction of EPMC with the amino acid residues in the binding site was shown in ball and stick (right). The different types of interactions are represented by different colors mentioned in the interactions color panel.

Supplementary Table S1. Drug likeness properties of EPMC by Swiss ADME web server.

Physicochemical properties of EPMC	
Chemical formula	$C_{12}H_{14}O_3$
Molecular weight (MW)	206.24 g/mol
Topological surface area (TPSA)	35.53 $Å^2$
Number of H-bond acceptors (nOHNH)	3
Number of H-bond donors (nON)	0
Water partition coefficient (WLOGP)	2.16
Number of rotatable bonds (nrotb)	5

Supplementary Table S2. Toxicity properties of EPMC by ProTox-II web server.

Toxicity	Prediction	Probability
Hepatotoxicity	Inactive	0.66
Cytotoxicity	Inactive	0.86
Carcinogenicity	Inactive	0.62
Mutagenicity	Inactive	0.66
Immunotoxicity	Inactive	0.69
